# Location Has Prognostic Impact on the Outcome of Colorectal Mucinous Adenocarcinomas

**DOI:** 10.3390/cancers16010147

**Published:** 2023-12-28

**Authors:** Matthew G. K. Benesch, Erek D. Nelson, Shalana B. L. O’Brien

**Affiliations:** Department of Surgical Oncology, Roswell Park Comprehensive Cancer Center, Buffalo, NY 14263, USA; erek.nelson@roswellpark.org (E.D.N.); shalana.obrien@roswellpark.org (S.B.L.O.)

**Keywords:** cancer, chemotherapy, colloid adenocarcinomas, epidemiology, histopathology, radiotherapy, surgery, signet ring cell adenocarcinomas, survival outcomes

## Abstract

**Simple Summary:**

Mucinous adenocarcinomas are an uncommon tumor type that arise primarily in the large intestine, characterized by the overexpression of a jelly-like substance called mucin outside their cells. In previous work, we showed that in the colon, overall survival outcomes were similar to regular adenocarcinomas, but significantly worse in the rectum. In this investigation, we show that these cancers are more prevalent in the right colon, where survival outcomes are equivalent or slightly better across all ages and stages. However, when mucinous adenocarcinomas are located more distally in the left colon and rectum, respectively, outcomes are progressively worse than conventional adenocarcinomas, up to 1.3-fold, even after correction for sex, tumor grade, stage at presentation, and cancer therapy. These findings suggest that mucinous adenocarcinomas have an intrinsically worse tumor biology in the distal colon and rectum. Further investigations into understanding this behavior could have treatment implications for improving patient outcomes.

**Abstract:**

Mucinous (colloid) adenocarcinomas (MAs) are a rare histological subtype of tumors defined by extracellular mucin comprising more than 50% of the tumor. These tumors are on a continuum of mucin-producing malignancies with signet ring cell adenocarcinomas (SRCCs), which instead produce intracellular mucin. Mucin-containing cancers occur primarily in the stomach and colon, where for SRCCs, outcomes are relatively worse in the proximal stomach and the rectum. It is not known if MAs have similar outcomes. In this study, we use the Surveillance, Epidemiology, and End Results (SEER) database to examine the effects of tumor localization, age, sex, and stage on colorectal and gastric cancer outcomes for MAs. For right colon cancers, MAs are more common, particularly in females, and have slightly better or equivalent outcomes across all stages and ages compared to conventional adenocarcinomas, but outcomes are progressively worse compared to conventional adenocarcinomas for left colon and rectal cancers. Unlike SRCCs, MAs have similar outcomes to conventional adenocarcinomas in all stomach locations. Overall, these results suggest that MAs have an intrinsically different tumor biology in the left colon and rectum that promotes pathogenesis. Decoding this phenomenon could lead to more effectively tailored patient treatment regimens.

## 1. Introduction

Mucinous adenocarcinomas (MAs), also called colloid carcinomas, are rare malignancies where extracellular mucin composes at least 50% of the tumor [1]. As a complex glycoprotein, mucin functions in many inflammatory and wound-healing processes [2]. In cancer, however, mucin expression and deregulation can shelter malignant cells from the immune system and disrupt physiological cell–cell junctions [3]. This promotion of anchorage-independent growth enables the evolution of sufficient cellular plasticity to instigate cellular invasion and metastasis [4]. A closely related rare histology is the signet ring cell adenocarcinoma (SRCC), where mucin is instead overexpressed intracellularly in greater than 50% of the tumor cells [5]. In many MAs, a signet cell component is present, and vice versa. Therefore, these cancers are formally defined either as MAs or SRCCs depending on the cellular location of the majority of the tumor mucin, while in reality these cancer types exist along a continual spectrum [5].

Because these tumors comprise 0.5–1.5% of all solid tumors, these cancers are largely excluded from prospective clinical trials, and most of our knowledge of their behavior comes either from small retrospective series, or national cancer databases that typically lack granular patient data. In recent years, we have published systematic studies on the epidemiology of both SRCCs and MAs across all major tumor sites [6,7]. Nearly 80% of all SRCCs and 50% of MAs arise in either the stomach or the large intestine [6,7]. Of all gastric cancers, SRCCs comprise 17% of cases and MAs comprise about 2% of cases, whereas for colorectal cancers, SRCCs comprise about 1% of cases and MAs comprise under 9% of cases [6,7]. Given such low percentages, population-level cancer data are required to make meaningful interpretations of the effects of clinicoepidemiological factors such as location age, sex, and disease stage on disease outcomes [8]. We previously used the Surveillance, Epidemiology, and End Results (SEER) database to conduct such an analysis for SRCCs [9]. In this study, we showed that for early onset localized and regional gastric cancers, SRCCs have the same overall risk of mortality compared to conventional adenocarcinomas, but over the age of 50 years, SRCCs have worse outcomes across all stages [9]. Gastric SRCCs are 2–3-fold more likely in younger patients, and more heavily favor the distal stomach [9]. Across all ages, stages, and locations, colorectal SRCCs have worse outcomes [9]. SRCCs favor the right colon, but outcomes are significantly worse for the left colon and rectal cancers [9]. Relative to adenocarcinomas, colorectal SRCCs have the worst outcomes in younger patients [9]. With these findings, we then re-examined our previous study on MA epidemiology, where we published that the overall survival rate for MAs of the colon was the same when compared to conventional adenocarcinomas, but the overall survival rate for rectal MAs was about 1.3-fold worse [7]. 

In this study, we use the SEER database to provide a standardized characterization of the outcomes of both colorectal and gastric MAs, by anatomical location, age [early onset (<50 years old) or late onset (≥50 years old)], sex, and stage at presentation. These outcomes are then compared to conventional adenocarcinomas. We have employed the same analysis techniques as we did to study these effects in colorectal and gastric SRCCs [9], thereby allowing readers to directly compare and contrast MAs and SRCCs to each other and to conventional adenocarcinomas.

## 2. Materials and Methods

### 2.1. Patient Selection

The National Cancer Institute’s SEER database (Bethesda, MD, USA) comprised from 18 SEER cancer registries was employed using data from 1992 to 2016, as previously described [6,7]. Data release from the SEER database does not require informed patient consent or review by an institutional review board. The SEER database was accessed and searched in compliance with signed user agreements. Exclusion criteria and all variables were defined previously [6,7].

### 2.2. Statistical Analysis

All selected data from SEER cancer registries were imported into Stata 15.1 (StataCorp LLC, College Station, TX, USA) for statistical analyses. A complete case analysis was completed after variable definition as described previously, resulting in an exclusion of about 1% of cases [6,7]. We then excluded cases for which tumors were not localizable (2.7% of all cases after complete case analysis for colorectal cancers, and 20.7% for gastric cancers, with proportional exclusion rates to both MAs and conventional adenocarcinomas for both histologies). The resulting ICD-O-3 codes used for patient selection are detailed in Table S1.

Baseline patient characteristics were compared with the *t* and *χ*^2^ tests for continuous and categorical variables, respectively. Univariate and multivariable Cox proportional hazard regression analyses were used to determine the association of mortality with cancer histology type, adjusting for age, sex, race, detection stage, grade differentiation, surgery, radiotherapy, and chemotherapy. All hazard ratios were calculated with 95% confidence intervals. The use of surgery, radiotherapy, and chemotherapy as treatment variables was treated as binary. All *p*-values were two-sided, with a threshold of 0.05 to determine statistical significance. Survival curves were plotted using the Kaplan–Meier method, with *p*-values for survival curves generated using the log-rank test. Graphs were plotted using Origin Pro 2022 (OriginLab Corporation, Northampton, MA, USA). Using SEER 18 (2000–2018) data with SEER*Stat 8.4.2 (Surveillance Research Program, National Cancer Institute, Calverton, MD, USA), incidence rates were calculated with age adjusted to the 2000 United States standard population with the age variable recode <1-year-old. Cause-specific survival and relative survival were both age standardized to the International Cancer Survival Standard 1-Age 15+ variable via the actuarial method, and Ederer II cumulative expected method for relative survival. 

## 3. Results

### 3.1. Frequency, Adjusted Mortality, and Survival Trends for Colorectal Mucinous Adenocarcinomas

For tumors that are localizable to regions of the large intestine, 54% of MAs reside in the right colon, of which nearly 24% are in the cecum, and 6.4% in the appendix (Table 1). This compares to only 33.4% of conventional adenocarcinomas arising in the right colon, with 16.0% in the cecum and only 0.4% in the appendix (Table 1). Conversely, only 17.4% of tumors arise in the rectal region, versus 30.3% for conventional adenocarcinomas (Table 1).

We next repeated the frequency distribution analysis dichotomized by sex and age (<50 years old, ≥50 years old) (Table 2). There is a noticeable bias towards right colon MAs in older females. In early onset cancer (<50 years of age), MAs are found in the right colon 47.2% of the time in females (vs. 46.2% for males), and in the ≥50-year-old group, this increases to 59.9% in females (vs. 47.2% for males). Conventional adenocarcinomas show no sex bias in males, having comprised about 21% of all cancers in both age groups, with an increase to 39.8% from 30.1% between older and younger females, respectively. Regarding the disease state at presentation, females under 50 years old present with distant disease in the right colon 40.9% of the time, as opposed to 31.4% of the time with adenocarcinomas. For left colon and rectal tumors, the percentage breakdown of the stage at presentation is comparable between sex and age groups (Table 2). With respect to incidence rates, the ratio between right colon and rectal cancers for conventional adenocarcinomas is about 0.5–0.6 for both males and females under 50 years old, compared to 1.8–2.4 for MAs. In the over 50-year-old group, for conventional adenocarcinomas, this ratio increased to about 1 for males, and 1.7 for females, and for MAs, this ratio increased to 2.7 for males, and up to 4.8 for females (Table 2). 

The results of the multivariable analyses show that when looking at all sites, MAs and conventional adenocarcinomas had essentially equivalent outcomes (Table 3). However, when the results are re-examined by location, patients with right colon MAs have better survival outcomes compared to patients with conventional adenocarcinomas (HR mortality about 0.9), which then flip for the left colon for a uniform HR of about 1.1–1.2 and increases slightly to 1.2–1.3 in the rectal region, with a maximal mortality HR of 1.40 (1.27–1.54) in the rectum in patients under 50 years old (Table 3).

Tables S2 and S3 compare mortality hazard ratios for individual sites relative to the transverse colon by conventional adenocarcinoma and MA groupings. The highest mortality HR for conventional adenocarcinomas are in the rectum of patients greater than 50 years old at 1.24 (1.20–1.27). For MAs, this instead increases to 1.45 (1.43–1.58), and the HR for rectal MAs against right colon MAs approaches 1.6 (Table S3).

**Figure 1:**
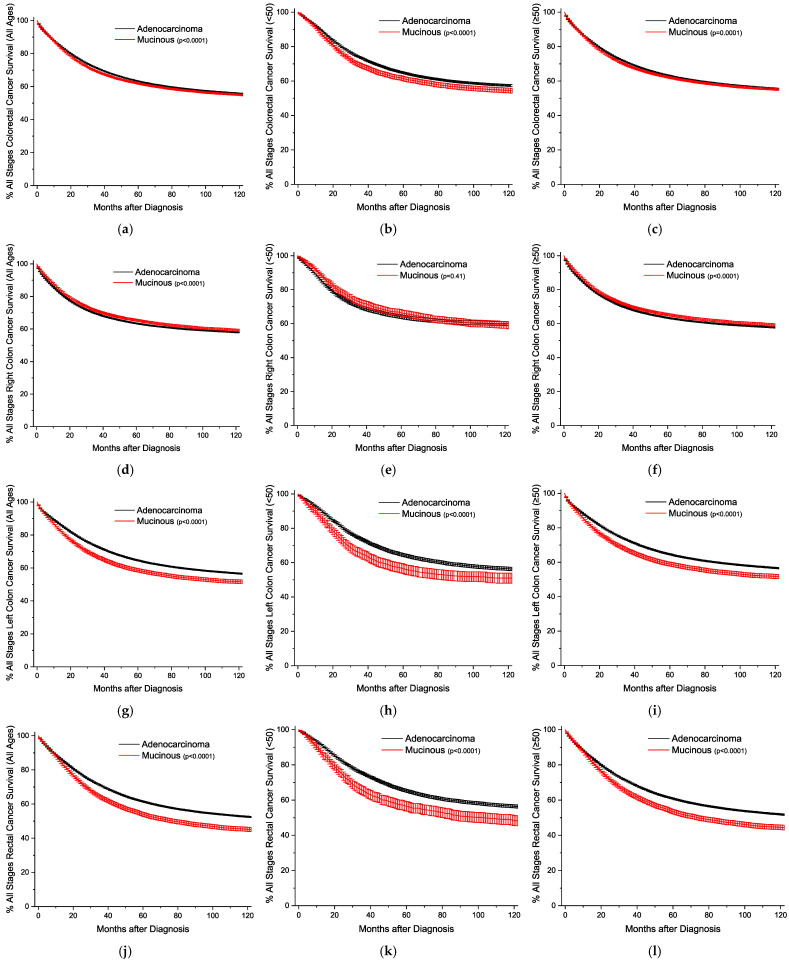
Kaplan–Meier survival curves for all stages of colorectal cancer, shown with 95% confidence intervals. (**a**) All locations, all ages. (**b**) All locations, age < 50. (**c**) All locations, age ≥ 50. (**d**) Right colon, all ages. (**e**) Right colon, age < 50. (**f**) Right colon, age ≥ 50. (**g**) Left colon, all ages. (**h**) Left colon, age < 50. (**i**) Left colon, age ≥ 50. (**j**) Rectal, all ages. (**k**) Rectal, age < 50. (**l**) Rectal, age ≥ 50. *p*-values between curves generated using log-rank test.

**Figure 2:**
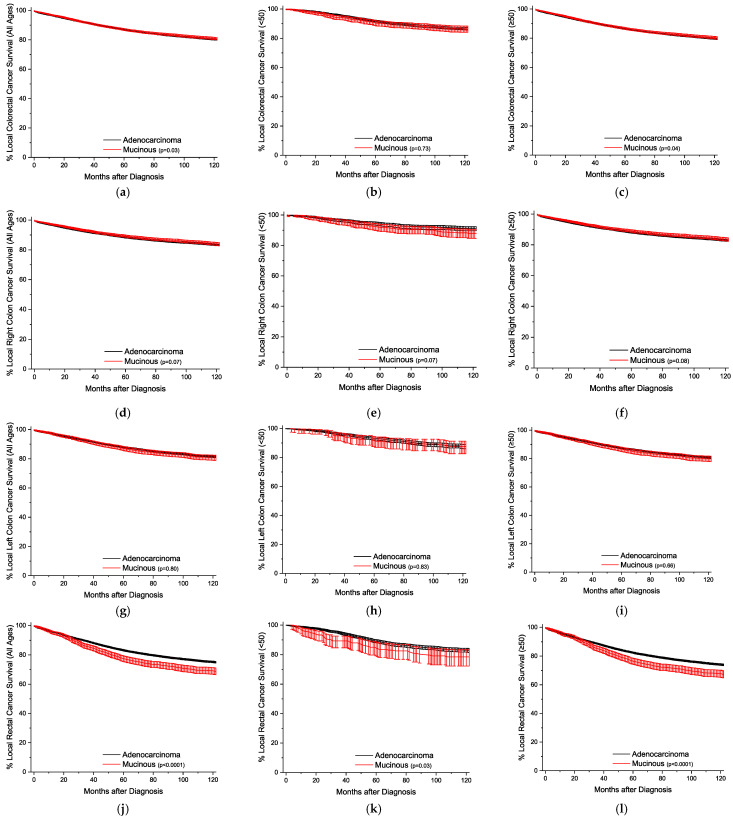
Kaplan–Meier survival curves for local colorectal cancer, shown with 95% confidence intervals. (**a**) All locations, all ages. (**b**) All locations, age < 50. (**c**) All locations, age ≥ 50. (**d**) Right colon, all ages. (**e**) Right colon, age < 50. (**f**) Right colon, age ≥ 50. (**g**) Left colon, all ages. (**h**) Left colon, age < 50. (**i**) Left colon, age ≥ 50. (**j**) Rectal, all ages. (**k**) Rectal, age < 50. (**l**) Rectal, age ≥ 50. *p*-values between curves generated using log-rank test.

**Figure 3:**
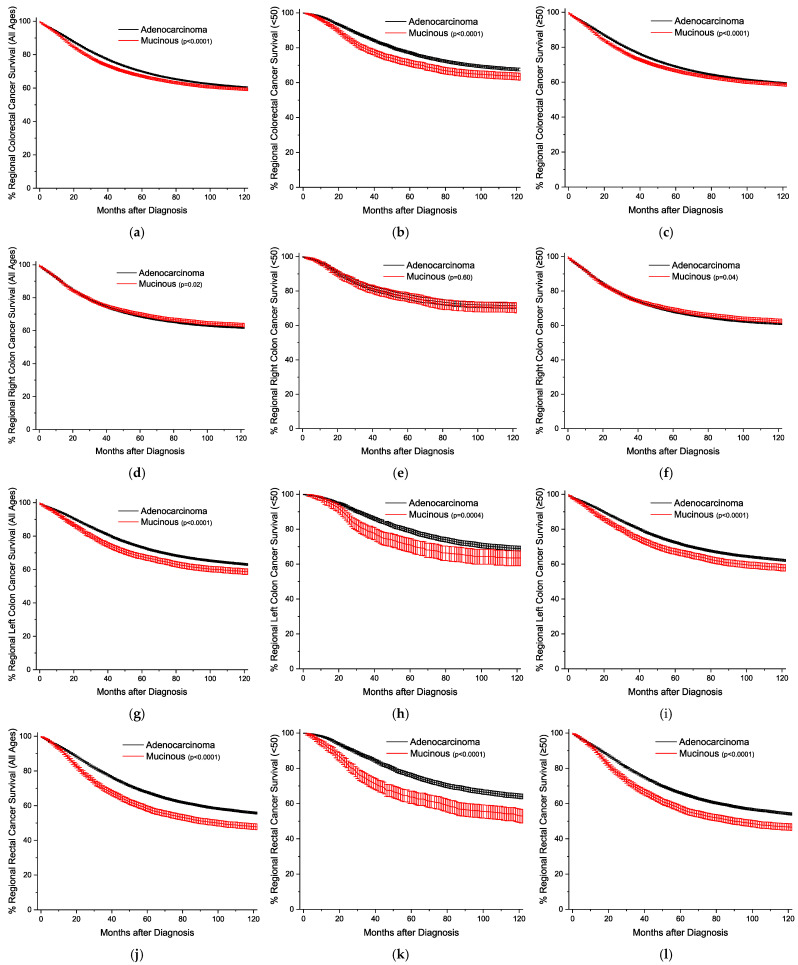
Kaplan–Meier survival curves for regional colorectal cancer, shown with 95% confidence intervals. (**a**) All locations, all ages. (**b**) All locations, age < 50. (**c**) All locations, age ≥ 50. (**d**) Right colon, all ages. (**e**) Right colon, age < 50. (**f**) Right colon, age ≥ 50. (**g**) Left colon, all ages. (**h**) Left colon, age < 50. (**i**) Left colon, age ≥ 50. (**j**) Rectal, all ages. (**k**) Rectal, age < 50. (**l**) Rectal, age ≥ 50. *p*-values between curves generated using log-rank test.

**Figure 4:**
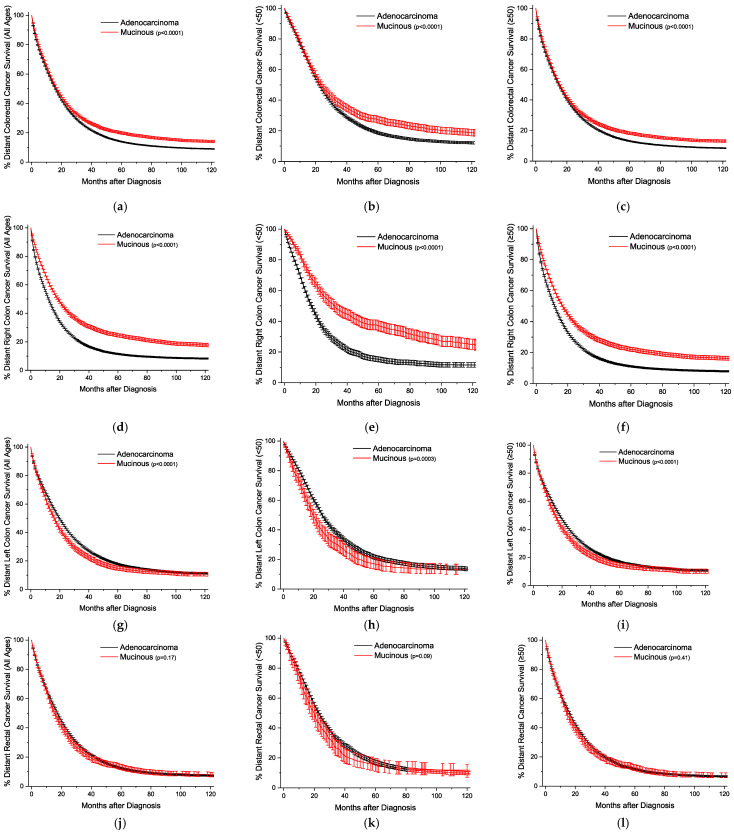
Kaplan–Meier survival curves for distant colorectal cancer, shown with 95% confidence intervals. (**a**) All locations, all ages. (**b**) All locations, age < 50. (**c**) All locations, age ≥ 50. (**d**) Right colon, all ages. (**e**) Right colon, age < 50. (**f**) Right colon, age ≥ 50. (**g**) Left colon, all ages. (**h**) Left colon, age < 50. (**i**) Left colon, age ≥ 50. (**j**) Rectal, all ages. (**k**) Rectal, age < 50. (**l**) Rectal, age ≥ 50. *p*-values between curves generated using log-rank test.

The Kaplan–Meier survival curves are plotted by age and location, sorted by the stage of detection. Figure 1 presents the plots with all stages grouped together. The overall survival trends are essentially identical, but statistically worse for the MA (Figure 1a–c). For all stages, survival for right colon cancer is statistically equivalent for the under-50-year-old group, and slightly better for the older group (Figure 1d–f). Across both age groups for left colon and rectal cancers, respectively, MA survival is progressively worse than conventional adenocarcinoma survival (Figure 1g–l). This style of analysis was again repeated for cancers detected at the local stage (Figure 2), and outcomes are worse for MAs only in rectal cancers (Figure 2j–l). For regional disease (Figure 3), the survival patterns are identical to those in the overall stages in Figure 1. For distant disease at presentation (Figure 4), right colon MA patients have a very significant survival advantage (Figure 4d–f), with a nearly doubled median survival time compared to conventional adenocarcinoma patients [cause-specific survival (CSS) 20.0 vs 11.9 months, relative survival (RS) 19.7 vs. 11.5 months] (Tables S4 and S5). The outcomes for distal left colon and rectal patients are equally dismal with nearly identical survival curves (Figure 4g–l), and medial survival times on the order of 15–20 months (Tables S4 and S5). Table S4 provides the CSS with 95% CIs for 1, 2, 5, and 10 years by stage at presentation, and Table S5 provides the same data for RS.

### 3.2. Frequency, Adjusted Mortality, and Survival Trends for Gastric Mucinous Adenocarcinomas

For localizable tumors, the distribution between the proximal and distal stomach is similar, with a slight increase for MAs in the distal stomach over conventional adenocarcinomas (37.6% versus 33.3%) (Table S6). Across both age groups and histologies, proximal tumors are more likely in males compared to females (about 70% versus 50%) (Table S7). Also, the mortality HR is the same in both the proximal and distal stomachs in both age groups when comparing MAs to conventional adenocarcinomas (Table S8). By a similar degree to conventional gastric adenocarcinomas, the mortality HR for distal tumors is slightly better (~0.7–0.8) than proximal tumors (Tables S9 and S10). Across all stages and ages, MAs and conventional adenocarcinomas have overlapping Kaplan–Meier survival curves (Figure S1). The CSS and RS tables by disease stage in both the proximal and distal stomach in both age groups, with 1, 2, 5, and 10-year and median survivals, are provided for reference (Tables S11 and S12).

## 4. Discussion

Colorectal MAs comprise half of all MAs [7], and this study represents the most comprehensive analysis of the histopathological characteristics by anatomical location. Nuances in the presentation and outcomes of colorectal MAs attributable to age, sex, stage, and location require numbers only available through population-level registry data. This paper, along with its sister companion publication on SRCCs [9], allows for a direct comparison across the spectrum of both colorectal (and gastric) SRCCs and MAs, with conventional adenocarcinomas. Consistent with our findings, the literature has reported colorectal MAs to be more common in the proximal colon and more common in younger patients and the female sex compared to conventional adenocarcinomas [10,11]. This study adds to this body of knowledge, because the literature reports conflicting findings regarding the prognosis of colorectal MA patients, often because studies are underpowered to detect differences based on location and demographic characteristics [12].

**Figure 5:**
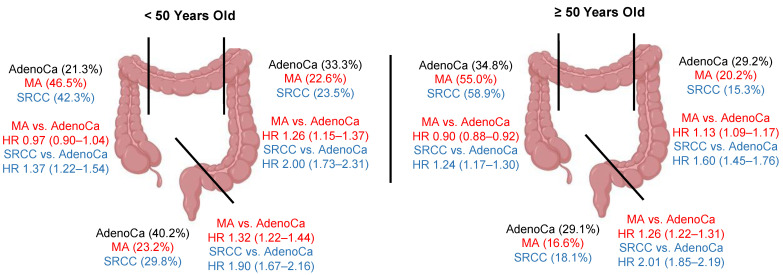
Graphical summary of key findings. Percent distribution of each cancer histology (black, conventional adenocarcinoma; red, mucinous adenocarcinoma; blue, signet ring cell adenocarcinoma) for the right colon, left colon, and rectal region. Mortality hazard ratios compared to conventional adenocarcinomas, with 95% confidence intervals in brackets. AdenoCa, conventional adenocarcinoma; MA, mucinous adenocarcinoma; SRCC, signet ring cell adenocarcinoma; HR, hazard ratio.

Colorectal MAs have both unique and common characteristics as SRCCs when compared to conventional adenocarcinomas. Both MAs and SRCCs are preferentially found in the right colon (54.0% vs. 55.3%), compared to 33.4% for conventional adenocarcinomas [9]. Both cancer histologies are even further biased towards the right colon for older females, accounting for 59.9% of all colorectal MAs in this demographic, and 65.1% for SRCCs, compared to just 39.8% in conventional adenocarcinomas [9]. Estrogen levels have been proposed to be protective against the development of right-sided colon cancers, a phenomenon which is diminished with menopause [13]. While the probability of presenting with distant disease is higher in the younger (<50 year old) group compared to the older demographic (≥50 year old) for all three histologies, there is again a stepwise percentage increase for both sexes (male/female): conventional adenocarcinoma (27.4%/31.4%), MA (31.5%/40.9%), and SRCC (44.6%/53.0%) [9]. Patients with right colon MAs have either equivalent or slightly better overall survival outcomes than patients with conventional adenocarcinomas, whereas for right colon SRCCs, the multivariable adjusted mortality HR is 1.28 (1.22–1.34). The reason for this finding is not clear. It is possible that right- and left-sided colon cancers have different mutation profiles for the same histology. For example, *BRAF* and *KRAS* mutations are much more common in right colon cancers, compared to *APC* and *TP53* mutations in left-sided colon cancers across different pathological histologies [14,15]. While survival trends are worse in the left colon/rectum compared to the right colon for both MAs and SRCCs, there is a clear stepwise increase in the mortality HR compared to conventional adenocarcinomas: MAs [left colon 1.16 (1.12–1.20), rectal 1.28 (1.34–1.32)], SRCCs [left colon 1.78 (1.64–1.92), and rectal 2.10 (1.96–2.25)] [9]. The highlights of these findings are summarized graphically in Figure 5. Finally, while colorectal malignancies make up the majority of MAs, gastric cancer comprises the bulk of SRCCs in a 2:1 ratio followed by colorectal cancer [6,7]. However, unlike SRCCs, which have worse outcomes relative to conventional adenocarcinomas across all stages, particularly in patients ≥50 years old [9], gastric MAs show no meaningful demographic or clinical outcome differences compared to conventional gastric adenocarcinomas. Taken together, these findings suggest that mucin-overproducing malignancies such as MAs and SRCCs exhibit unique patterns of pathology in the large intestine. The implication here is that these cancers likely have unique tumor biology characteristics that, if decoded, could be exploited by novel treatment regimens.

An understanding of this tumor biology is emerging in the literature. A recent retrospective analysis of microsatellite instability in 502 colorectal cases demonstrated an increased correlation with the female sex (*p* = 0.026), right colon disease (*p* = 0.01), and MA or adenocarcinoma with a mucinous component (*p* < 0.01) [16]. The proposed mechanisms for this association with female sex include changes in hormonal levels with menopause, endogenous hormonal exposure, microbiome influences, and sex-based differences in access to screening modalities [17,18,19]. Similarly, increased colorectal SRCC pathogenesis has been correlated to both increased rates of *BRAF* mutations and microsatellite instability [20]. For patients with microsatellite instability, recent advances in immunotherapy, and the adoption of such treatments into standard-of-care algorithms for patients with loss of mismatch-repair protein expression, are a promising breakthrough towards improving outcomes [21,22].

The detection of the disease and definitive surgical treatments at earlier disease stages are more effective than novel therapies for improving patient outcomes. The incidence of early-onset (<50 years old) colorectal cancer is on the rise and expected to double by 2030, yet most colorectal cancer-screening programs do not target this demographic [23,24]. The majority of these early-onset colorectal cancers arise in patients 40-49 years, with a left-sided predominance, but there is increased mortality with the right-sided disease [25]. An analysis of patients in this population using a large tertiary center database was performed, and it was found that MAs and SRCCs were three-fold more likely to present in the right colon compared to the left, with a lower overall 5-year survival [25]. These findings have been replicated in other centers [26,27,28]. It is predicted that population-level colorectal screening beginning at the age of 40 could have tangible benefits in decreasing disease burden through increased detection and management of asymptomatic cases or premalignant lesions [29]. 

There are several limitations in this study. Although this project employs a well-validated population-level database with a rigorous quality-improvement methodology [30], the research is retrospective in nature. Regarding limitations to the generalizability of the results, this study examines an American population that is predominantly white (~80%), whereas racial minorities account for close to 40% of the actual population distribution [31]. This study also does not consider financial or other social determinants of health on cancer outcomes. Treatment modalities and staging has also changed considerably over the near 30 years of patient data that are encompassed in this study, and as such, treatment variables have only been recorded as binary, and staging information was captured in broad categories. To study the more nuanced effects of the pathological and treatment influence on subgroup outcomes defined by age or location, more granular data as defined by TNM staging criteria, and precise treatment regimens and sequences, are required. Nevertheless, the robust characterization of the effects of tumor localization on the outcomes of MAs would not necessarily be feasible given the prevalence of this rare histological subtype.

## 5. Conclusions

This investigation represents among the largest studies of the demographics and histopathological characteristics of colorectal and gastric MAs by anatomical location, and by utilizing the same study design as our previous work on colorectal and gastric SRCCs, these publications will enable readers to utilize a standardized overview of mucin-producing gastrointestinal malignancies. Such a baseline characterization is necessary to propose and systematically design appropriately powered studies necessary to decode the underlying tumor biology of these rare histologies. In particular, understanding the connections among sex, age, location, and mutational changes that lead to these rare malignancies are all areas of investigation. Decoding this biology, and the association–causation relationship between mutational burden and histology will be required to design histology-tailored treatment and surveillance programs for the improvement of patient prognosis beyond the shortcomings of standard-of-care algorithms that may not apply well outside of conventional tumors.

## Figures and Tables

**Table 1:** Distribution of colorectal conventional adenocarcinoma and mucinous adenocarcinoma by location.

Colorectal Location	Adenocarcinoma	Mucinous
**All Sites**	393,879 (100)	50,776 (100)
**Right Colon**	131,482 (33.4)	27,412 (54.0)
Appendix	1761 (0.4)	3266 (6.4)
Cecum	62,888 (16.0)	12,079 (23.8)
Ascending Colon	51,752 (13.1)	9416 (18.5)
Hepatic Flexure	15,081 (3.8)	2651 (5.2)
**Transverse Colon**	26,492 (6.7)	4093 (8.1)
**Left**	116,632 (29.6)	10,426 (20.5)
Splenic Flexure	10,985 (2.8)	1450 (2.9)
Descending Colon	18,161 (4.6)	2019 (4.0)
Sigmoid Colon	87,486 (22.2)	6957 (13.7)
**Rectal**	119,273 (30.3)	8845 (17.4)
Rectosigmoid	37,657 (9.6)	2797 (5.5)
Rectum	81,616 (20.7)	6048 (11.9)

*p* < 0.05 for all comparisons between conventional adenocarcinomas and mucinous adenocarcinomas among right colon, transverse colon, left colon, and rectal comparisons.

**Table 2:** Distribution of colorectal cancer by localization, dichotomized by sex and age groupings.

Colorectal Location	Adenocarcinoma				Mucinous			
Sex	Male		Female		Male		Female	
Age (Years)	<50	≥50	<50	≥50	<50	≥50	<50	≥50
**All Sites**								
*N* (%)	22,579 [11.1]	181,541 [88.9]	19,470 [10.3]	170,289 [89.7]	3607 [14.8]	20,814 [85.2]	2657 (10.1)	23,698 [89.9]
	(100)	(100)	(100)	(100)	(100)	(100)	(100)	(100)
Stage								
In Situ	101 (0.4)	1292 (0.7)	95 (0.5)	1091 (0.6)	4 (0.1)	9 (0.1)	2 (0.1)	14 (0.1)
Localized	5263 (23.3)	58,867 (32.4)	4618 (23.7)	55,919 (32.8)	765 (21.2)	6046 (29.0)	523 (19.7)	7204 (30.4)
Regional	10,294 (45.6)	75,339 (41.5)	8895 (45.7)	72,384 (42.5)	1724 (47.8)	9624 (46.2)	1132 (42.6)	10,925 (46.1)
Distant	6410 (28.4)	39,790 (21.9)	5545 (28.5)	34,201 (20.1)	1057 (29.3)	4826 (23.2)	951 (35.8)	5207 (22.0)
Unstaged	511 (2.3)	6253 (3.4)	317 (1.6)	6694 (3.9)	57 (1.6)	309 (1.5)	49 (1.8)	348 (1.5)
Incidence	4.43 (4.38–4.49)	104.8 (104.4–105.3)	3.80 (3.75–3.85)	75.0 (74.7–75.3)	0.65 (0.63–0.67)	11.5 (11.4–11.7)	0.48 (0.46–0.50)	10.0 (9.9–10.2)
**Right**								
*N* (%)	4780 (21.2)	54,707 (30.1)	4171 (21.4)	67,824 (39.8)	1665 (46.2)	10,302 (49.5)	1253 (47.2)	14,192 (59.9)
Stage								
In Situ	23 (0.5)	401 (0.7)	19 (0.5)	417 (0.6)	4 (0.2)	5 (0.1)	2 (0.2)	8 (0.1)
Localized	1188 (24.9)	18,183 (33.2)	1010 (24.2)	23,069 (34.0)	374 (22.5)	3174 (30.8)	280 (22.3)	4571 (32.2)
Regional	2206 (46.2)	22,580 (41.3)	1787 (42.8)	28,555 (42.1)	734 (44.1)	4565 (44.3)	432 (34.5)	6388 (45.0)
Distant	1310 (27.4)	12,093 (22.1)	1308 (31.4)	13,618 (20.1)	525 (31.5)	2437 (23.7)	513 (40.9)	3063 (21.6)
Unstaged	53 (1.1)	1450 (2.7)	47 (1.1)	2165 (3.2)	28 (1.7)	121 (1.2)	26 (2.1)	162 (1.1)
Incidence	0.92 (0.90–0.95)	34.0 (33.7–34.2)	0.80 (0.78–0.82)	30.8 (30.6–31.0)	0.30 (0.29–0.32)	5.93 (5.82–6.04)	0.24 (0.22–0.25)	6.06 (5.97–6.16)
**Transverse**								
*N* (%)	1188 (5.3)	11,318 (6.2)	1062 (5.5)	12,924 (7.6)	275 (7.6)	1522 (7.3)	201 (7.6)	2095 (8.8)
Stage								
In Situ	2 (0.2)	64 (0.6)	6 (0.6)	64 (0.5)	0 (0.0)	1 (0.1)	0 (0.0)	0 (0.0)
Localized	285 (24.0)	3750 (33.1)	244 (23.0)	4224 (32.7)	59 (21.5)	471 (30.9)	41 (20.4)	636 (30.4)
Regional	588 (49.5)	4983 (44.0)	475 (44.7)	5899 (45.6)	134 (48.7)	748 (49.1)	98 (48.8)	1041 (49.7)
Distant	297 (25.0)	2242 (19.8)	327 (30.8)	2360 (18.3)	82 (29.8)	291 (19.1)	62 (30.8)	394 (18.8)
Unstaged	16 (1.3)	279 (2.5)	10 (0.9)	10 (0.9)	0 (0.0)	11 (0.7)	0 (0.0)	24 (1.1)
Incidence	0.24 (0.23–0.25)	7.48 (7.36–7.60)	0.21 (0.20–0.22)	6.13 (6.03–6.22)	0.052 (0.046–0.058)	0.95 (0.91–1.00)	0.035 (0.031–0.041)	0.97 (0.93–1.01)
**Left**								
*N* (%)	6760 (29.9)	55,415 (30.5)	7205 (37.0)	47,252 (27.7)	763 (21.2)	4692 (22.5)	653 (24.6)	4318 (18.2)
Stage								
In Situ	39 (0.6)	453 (0.8)	36 (0.5)	325 (0.7)	0 (0.0)	3 (0.1)	0 (0.0)	2 (0.1)
Localized	1437 (21.3)	17,279 (31.2)	1593 (22.1)	14,719 (31.2)	169 (22.1)	1298 (27.7)	118 (18.1)	1207 (28.0)
Regional	2922 (43.2)	22,701 (41.0)	3250 (45.1)	20,191 (42.7)	331 (43.4)	2045 (43.6)	293 (44.9)	1942 (45.0)
Distant	2262 (33.5)	13,432 (24.2)	2261 (31.4)	10,464 (22.1)	257 (33.7)	1303 (27.8)	233 (35.7)	1108 (25.7)
Unstaged	100 (1.5)	1550 (2.8)	65 (0.9)	1553 (3.3)	6 (0.8)	43 (0.9)	9 (1.4)	59 (1.4)
Incidence	1.33 (1.30–1.36)	30.4 (30.2–30.6)	1.41 (1.38–1.44)	19.9 (19.7–20.0)	0.14 (0.13–0.15)	2.50 (2.43–2.57)	0.11 (0.10–0.12)	1.76 (1.71–1.82)
**Rectal**								
*N* (%)	9851 (43.6)	60,101 (33.1)	7032 (36.1)	42,289 (24.8)	904 (25.1)	4298 (20.6)	550 (20.7)	3093 (13.1)
Stage								
In Situ	37 (0.4)	374 (0.6)	34 (0.5)	285 (0.7)	0 (0.0)	0 (0.0)	0 (0.0)	4 (0.1)
Localized	2353 (23.9)	19,655 (32.7)	1771 (25.2)	13,907 (32.9)	163 (18.0)	1103 (25.7)	84 (15.3)	790 (25.5)
Regional	4578 (46.5)	25,075 (41.7)	3383 (48.1)	17,739 (41.9)	525 (58.1)	2266 (52.7)	309 (56.2)	1554 (50.2)
Distant	2541 (25.8)	12,023 (20.0)	1649 (23.4)	7759 (18.3)	193 (21.3)	795 (18.5)	143 (26.0)	642 (20.8)
Unstaged	342 (3.5)	2974 (4.9)	195 (2.8)	2599 (6.1)	23 (2.5)	134 (3.1)	14 (2.5)	103 (3.3)
Incidence	1.94 (1.90–1.98)	32.9 (32.7–33.2)	1.39 (1.36–1.42)	18.2 (18.1–18.4)	0.16 (0.15–0.17)	2.16 (2.09–2.22)	0.10 (0.09–0.11)	1.25 (1.21–1.30)

*p* < 0.05 for all comparisons between conventional adenocarcinomas and mucinous adenocarcinomas. [ ] indicates percentages across the age groups within each sex; ( ) indicates percentages within each column. Incidence rates are expressed per 100,000.

**Table 3:** Derived univariate and multivariable Cox proportional hazard ratios of mortality for colorectal mucinous adenocarcinomas versus conventional adenocarcinomas.

Colorectal Location	Mucinous vs. Adenocarcinoma (All Ages)		Mucinous vs. Adenocarcinoma (Age < 50)		Mucinous vs. Adenocarcinoma (Age ≥ 50)	
HR (95% CI)	Univariate	Multivariable	Univariate	Multivariable	Univariate	Multivariable
**All Sites**	1.04 (1.03–1.05)	1.02 (1.01–1.04)	1.13 (1.08–1.18)	1.05 (1.00–1.09)	1.03 (1.02–1.05)	1.01 (0.99–1.03)
**Transverse Colon**	1.03 (0.98–1.09)	1.04 (0.98–1.10)	1.16 (0.99–1.36)	1.12 (0.95–1.32)	1.02 (0.96–1.08)	1.02 (0.96–1.09)
**Right Colon**	0.94 (0.92–0.96)	0.89 (0.87–0.91)	0.97 (0.90–1.04)	0.82 (0.76–0.88)	0.94 (0.92–0.96)	0.90 (0.88–0.92)
Appendix	0.84 (0.77–0.93)	0.64 (0.57–0.71)	0.83 (0.68–1.01)	0.58 (0.46–0.74)	0.86 (0.77–0.96)	0.65 (0.57–0.73)
Cecum	0.92 (0.89–0.95)	0.94 (0.91–0.97)	0.97 (0.86–1.08)	0.98 (0.87–1.10)	0.91 (0.88–0.95)	0.93 (0.90–0.97)
Ascending Colon	0.95 (0.91–0.99)	1.03 (0.99–1.08)	1.07 (0.94–1.23)	1.19 (1.04–1.37)	0.94 (0.90–0.98)	1.02 (0.98–1.06)
Hepatic Flexure	0.91 (0.85–0.98)	1.04 (0.96–1.12)	0.72 (0.56–0.92)	0.93 (0.72–1.20)	0.94 (0.87–1.01)	1.04 (0.96–1.12)
**Left**	1.20 (1.16–1.23)	1.16 (1.12–1.20)	1.25 (1.15–1.36)	1.26 (1.15–1.37)	1.19 (1.15–1.23)	1.13 (1.09–1.17)
Splenic Flexure	1.04 (0.95–1.14)	1.02 (0.94–1.12)	1.00 (0.78–1.29)	1.02 (0.78–1.32)	1.05 (0.96–1.16)	1.02 (0.92–1.12)
Descending Colon	1.04 (0.96–1.12)	1.08 (0.99–1.17)	1.09 (0.91–1.32)	1.25 (1.04–1.51)	1.03 (0.95–1.13)	1.04 (0.95–1.13)
Sigmoid Colon	1.27 (1.22–1.32)	1.21 (1.16–1.25)	1.40 (1.26–1.55)	1.31 (1.18–1.45)	1.26 (1.20–1.31)	1.18 (1.13–1.23)
**Rectal**	1.23 (1.19–1.27)	1.28 (1.24–1.32)	1.32 (1.22–1.44)	1.32 (1.22–1.44)	1.22 (1.18–1.26)	1.26 (1.22–1.31)
Rectosigmoid	1.29 (1.21–1.36)	1.24 (1.17–1.31)	1.35 (1.16–1.56)	1.11 (0.95–1.30)	1.28 (1.20–1.36)	1.25 (1.17–1.33)
Rectum	1.21 (1.16–1.25)	1.29 (1.24–1.34)	1.32 (1.20–1.45)	1.40 (1.27–1.54)	1.19 (1.14–1.25)	1.26 (1.21–1.32)

*p* < 0.05 for all results unless confidence interval crosses 1. Multivariable adjustment corrected for sex, race, detection stage, grade differentiation, surgery, radiotherapy, and chemotherapy. HR: hazard ratios.

## Data Availability

Data release from the SEER database (https://seer.cancer.gov, accessed on 31 August 2023).

## Supplementary Materials

The following supporting information can be downloaded at: https://www.mdpi.com/article/10.3390/cancers16010147/s1. Table S1: Definitions of organ localization by ICD-O-3 codes within this study; Table S2: Derived univariate and multivariable Cox proportional hazard ratios of mortality for colorectal adenocarcinomas; Table S3: Derived univariate and multivariable Cox proportional hazard ratios of mortality for conventional colorectal mucinous adenocarcinomas; Table S4: Cause-specific survival of colorectal conventional adenocarcinomas and mucinous adenocarcinomas by age group and site; Table S5: Relative survival of colorectal conventional adenocarcinomas and mucinous adenocarcinomas by age group and site; Table S6: Distributions of gastric conventional adenocarcinomas and mucinous adenocarcinomas by location; Table S7: Distribution of gastric cancer by localization, dichotomized by sex and age groupings; Table S8: Derived univariate and multivariable Cox proportional hazard ratios of mortality for gastric mucinous adenocarcinomas versus conventional adenocarcinomas; Table S9: Derived univariate and multivariable Cox proportional hazard ratios of mortality for conventional gastric adenocarcinomas; Table S10: Derived univariate and multivariable Cox proportional hazard ratios of mortality for gastric mucinous adenocarcinomas; Figure S1: Kaplan–Meier survival curves for gastric cancer; Table S11: Cause-specific survival of gastric conventional adenocarcinomas and mucinous adenocarcinomas by age group and site; Table S12: Relative survival gastric conventional adenocarcinomas and mucinous adenocarcinomas by age group and site.
