# Supplementary material for: Location Has Prognostic Impact on the Outcome of Colorectal Mucinous Adenocarcinomas
Source: Cancers (Basel). 2023 Dec 28;16(1):147. doi: 10.3390/cancers16010147 (PMC10778219; doi:10.3390/cancers16010147)
Supplement: Supplementary file 1 [file cancers-16-00147-s001.zip › cancers-2761263-supplementary.pdf]

Supplementary Material

Location has Prognostic Impact on the Outcome of Colorectal Mucinous Adenocarcinomas

Table S1. Definitions of organ localization by ICD-O-3 codes within this study.

Colorectal*	Gastric**
**Right Colon**<br>Cecum – C18.0<br>Appendix – C18.1<br>Ascending Colon – C18.2<br>Hepatic Flexure of Colon – C18.3	**Proximal Stomach**<br>Cardia – C16.0<br>Fundus – C16.1<br>Body – C16.2
**Transverse Colon** – C18.4	**Distal Stomach**<br>Antrum – C16.3<br>Pylorus – C16.4
**Left Colon**<br>Splenic Flexure of Colon – C18.5<br>Descending Colon – C18.6<br>Sigmoid Colon – C18.7	
**Rectal**<br>Rectosigmoid junction – C19.9<br>Rectum – C20.9	

*Excluded colorectal codes are C18.8 (overlapping lesion of colon), C19.9 (colon, NOS).
**Excluded gastric codes are C16.5 (lesser curvature of stomach, NOS (not otherwise specified) not classifiable to C16.1-C16.4), C16.6 (greater curvature of stomach, NOS, not classifiable to C16.0-C16.4), C16.8 (overlapping lesion of stomach), and C16.9 (stomach, NOS).

Table S2. Derived univariate and multivariable Cox-proportional hazard ratios of mortality for conventional colorectal adenocarcinomas.

Colorectal Location	Adenocarcinoma (All Ages)		Adenocarcinoma (Age <50)		Adenocarcinoma (Age ≥50)	
**HR (95% CI)**	**Univariate**	**Multivariable**	**Univariate**	**Multivariable**	**Univariate**	**Multivariable**
**Transverse Colon**	1.00 (Reference)	1.00 (Reference)	1.00 (Reference)	1.00 (Reference)	1.00 (Reference)	1.00 (Reference)
**Right Colon**	1.07 (1.05-1.10)	1.01 (0.99-1.04)	1.02 (0.95-1.11)	1.03 (0.95-1.12)	1.08 (1.05-1.10)	1.01 (0.99-1.04)
Appendix	1.31 (1.21-1.42)	1.16 (1.07-1.26)	1.25 (1.05-1.49)	1.40 (1.16-1.67)	1.34 (1.23-1.47)	1.12 (1.03-1.23)
Cecum	1.18 (1.14-1.20)	1.05 (1.02-1.07)	1.07 (0.98-1.16)	1.00 (0.91-1.09)	1.18 (1.15-1.21)	1.05 (1.03-1.08)
Ascending Colon	0.95 (0.93-0.98)	0.96 (0.93-0.99)	0.93 (0.85-1.02)	1.02 (0.93-1.12)	0.95 (0.93-0.98)	0.96 (0.93-0.98)
Hepatic Flexure	1.07 (1.03-1.10)	1.03 (0.99-1.06)	1.10 (0.97-1.24)	1.10 (0.98-1.24)	1.06 (1.03-1.10)	1.02 (0.98-1.06)
**Left**	1.02 (1.00-1.05)	0.96 (0.94-0.98)	1.03 (0.95-1.11)	0.94 (0.87-1.02)	1.03 (1.01-1.06)	0.97 (0.95-1.00)
Splenic Flexure	1.10 (1.06-1.15)	1.05 (1.01-1.10)	1.00 (0.89-1.13)	1.02 (0.90-1.15)	1.12 (1.07-1.16)	1.05 (1.01-1.10)
Descending Colon	1.02 (0.99-1.06)	0.96 (0.93-0.99)	0.98 (0.89-1.09)	0.91 (0.82-1.01)	1.03 (0.99-1.06)	0.97 (0.93-1.00)
Sigmoid Colon	1.02 (1.00-1.05)	0.94 (0.92-0.97)	1.05 (0.97-1.13)	0.94 (0.87-1.02)	1.02 (1.00-1.05)	0.96 0.93-0.98)
**Rectal**	1.14 (1.12-1.17)	1.15 (1.12-1.18)	1.02 (0.95-1.10)	1.06 (0.98-1.15)	1.17 (1.14-1.20)	1.17 (1.14-1.20)
Rectosigmoid	1.10 (1.07-1.13)	1.05 (1.02-1.08)	1.05 (0.97-1.15)	1.00 (0.91-1.10)	1.11 (1.08-1.14)	1.07 (1.04-1.10)
Rectum	1.16 (1.14-1.19)	1.21 (1.18-1.24)	1.01 (0.93-1.09)	1.10 (1.00-1.21)	1.20 (1.17-.23)	1.24 (1.20-1.27)

$p < 0.05$ for all results unless confidence interval crosses 1. Multivariable adjustment corrected for sex, race, detection stage, grade differentiation, surgery, radiotherapy, and chemotherapy. HR, hazard ratios.

Table S3. Derived univariate and multivariable Cox-proportional hazard ratios of mortality for colorectal mucinous adenocarcinomas.

Colorectal Location	Mucinous (All Ages)		Mucinous (Age <50)		Mucinous (Age ≥50)	
**HR (95% CI)**	**Univariate**	**Multivariable**	**Univariate**	**Multivariable**	**Univariate**	**Multivariable**
**Transverse Colon**	1.00 (Reference)	1.00 (Reference)	1.00 (Reference)	1.00 (Reference)	1.00 (Reference)	1.00 (Reference)
**Right Colon**	0.98 (0.92-1.03)	0.89 (0.84-0.94)	0.86 (0.73-1.00)	0.80 (0.68-0.94)	0.99 (0.94-1.06)	0.91 (0.85-0.96)
Appendix	1.10 (1.02-1.19)	0.67 (0.61-0.74)	0.92 (0.77-1.10)	0.70 (0.56-0.88)	1.16 (1.06-1.27)	0.69 (1.20-1.31)
Cecum	1.04 (0.98-1.10)	0.95 (0.89-1.00)	0.89 (0.75-1.06)	0.88 (0.73-1.06)	1.06 (0.99-1.13)	0.96 (0.90-1.02)
Ascending Colon	0.88 (0.82-0.93)	0.96 (0.90-1.02)	0.86 (0.71-1.04)	1.10 (0.90-1.34)	0.88 (0.82-0.94)	0.95 (0.89-1.02)
Hepatic Flexure	0.94 (0.86-1.02)	1.01 (0.92-1.10)	0.68 (0.52-0.88)	0.94 (0.71-1.23)	0.98 (0.89-1.07)	1.02 (0.93-1.11)
**Left**	1.19 (1.12-1.27)	1.05 (0.99-1.12)	1.11 (0.94-1.31)	1.09 (0.92-1.29)	1.21 (1.13-1.29)	1.06 (0.99-1.14)
Splenic Flexure	1.11 (1.01-1.23)	1.01 (0.91-1.11)	0.87 (0.66-1.14)	0.96 (0.72-1.27)	1.16 (1.04-1.29)	1.03 (0.92-1.14)
Descending Colon	1.03 (0.94-1.12)	0.95 (0.86-1.04)	0.93 (0.74-1.16)	0.98 (0.77-1.23)	1.04 (0.94-1.15)	0.95 (0.85-1.05)
Sigmoid Colon	1.26 (1.18-1.35)	1.08 (1.01-1.16)	1.25 (1.05-1.49)	1.11 (0.93-1.34)	1.26 (1.18-1.35)	1.10 (1.02-1.18)
**Rectal**	1.37 (1.29-1.45)	1.32 (1.23-1.42)	1.16 (0.99-1.37)	1.12 (0.91-1.37)	1.41 (1.32-1.50)	1.38 (1.28-1.49)
Rectosigmoid	1.37 (1.27-1.47)	1.20 (1.10-1.30)	1.21 (0.99-1.49)	0.95 (0.75-1.22)	1.39 (1.28-1.51)	1.25 (1.14-1.37)
Rectum	1.37 (1.28-1.46)	1.39 (1.28-1.51)	1.14 (0.96-1.35)	1.21 (0.95-1.55)	1.41 (1.32-1.51)	1.45 (1.32-1.58)

$p < 0.05$ for all results unless confidence interval crosses 1. Multivariable adjustment corrected for sex, race, detection stage, grade differentiation, surgery, radiotherapy, and chemotherapy. HR, hazard ratios.

Table S4. Cause-specific survival of colorectal conventional adenocarcinomas and mucinous adenocarcinomas by age group and site.

Colorectal Location	All Patients		Age < 50 years		Age ≥50 years	
Cause-Specific Survival % (95% CI)	Adenocarcinoma	Mucinous	Adenocarcinoma	Mucinous	Adenocarcinoma	Mucinous
**All Sites**						
**Localized**						
1-year	95.1 (95.0-95.2)	95.7 (95.3-96.1)	99.0 (98.8-99.2)	98.4 (97.5-99.0)	94.7 (94.6-94.9)	95.4 (95.0-95.8)
2-year	92.5 (92.3-92.6)	93.1 (92.6-93.6)	97.7 (97.3-98.0)	96.4 (95.0-97.4)	92.0 (91.8-92.2)	92.8 (92.3-93.3)
5-year	85.8 (85.5-86.0)	86.3 (85.6-86.9)	92.1 (91.5-92.7)	90.8 (88.8-92.5)	85.2 (84.9-85.4)	85.8 (85.1-86.5)
10-year	79.5 (79.2-79.8)	80.2 (79.3-81.0)	86.6 (85.8-87.4)	85.9 (83.3-88.0)	78.8 (78.5-79.1)	79.6 (78.6-80.5)
Median (months)	-	-	-	-	-	-
**Regional**						
1-year	91.7 (91.5-91.8)	90.5 (90.1-91.0)	97.4 (97.2-97.7)	95.8 (94.9-96.6)	90.9 (90.7-91.0)	89.7 (89.2-90.2)
2-year	85.1 (84.9-85.3)	81.6 (81.0-82.2)	92.5 (92.0-92.9)	87.7 (86.2-89.0)	84.1 (83.8-84.3)	80.7 (80.0-81.3)
5-year	70.4 (70.1-70.7)	66.5 (65.8-67.3)	79.1 (78.4-79.7)	72.0 (70.0-73.8)	69.2 (68.9-69.5)	65.7 (64.9-66.5)
10-year	60.8 (60.5-61.1)	58.7 (57.8-59.5)	69.0 (68.1-69.8)	63.8 (61.6-65.9)	59.6 (59.2-59.9)	57.9 (57.0-58.8)
Median (months)	-	-	-	-	-	-
**Distant**						
1-year	57.9 (57.5-58.2)	61.6 (60.6-62.6)	74.2 (73.3-75.1)	75.7 (73.4-77.8)	55.1 (54.6-55.5)	58.5 (57.4-59.6)
2-year	37.3 (36.9-37.7)	40.7 (39.7-41.7)	50.4 (49.4-51.4)	51.8 (49.2-54.2)	35.1 (34.7-35.5)	38.3 (37.2-39.4)
5-year	14.2 (13.9-14.5)	20.7 (19.9-21.6)	19.7 (18.9-20.5)	28.8 (26.5-31.1)	13.2 (12.9-13.5)	18.9 (18.0-19.9)
10-year	8.5 (8.6-9.1)	14.5 (13.7-15.3)	12.5 (11.8-13.3)	20.3 (18.1-22.6)	8.2 (7.9-8.5)	13.2 (12.3-14.1)
Median (months)	16.1	17.8	24.2	25.2	14.6	16.2
**Right Colon**						
**Localized**						
1-year	95.1 (94.9-95.3)	95.9 (95.4-96.3)	99.1 (98.5-99.4)	99.3 (98.0-99.7)	94.9 (94.6-95.1)	95.6 (95.0-96.1)
2-year	92.6 (92.4-92.9)	93.7 (93.1-94.3)	97.5 (96.7-98.2)	97.0 (95.1-98.1)	92.4 (92.1-92.6)	93.4 (92.8-94.0)
5-year	87.1 (86.8-87.5)	88.5 (87.6-89.2)	93.8 (92.6-94.9)	91.6 (88.8-93.8)	86.7 (86.4-87.1)	88.2 (87.3-89.0)
10-year	82.2 (81.7-82.6)	83.2 (82.1-84.2)	90.4 (88.8-91.8)	86.6 (83.0-89.5)	81.7 (81.2-82.2)	82.9 (81.7-83.9)
Median (months)	-	-	-	-	-	-
**Regional**						
1-year	91.5 (89.3-89.9)	89.9 (89.2-90.5)	96.1 (95.4-96.8)	96.6 (95.2-97.6)	89.1 (88.8-89.4)	89.1 (88.4-89.8)
2-year	84.1 (81.4-82.2)	81.5 (80.7-82.3)	89.4 (88.2-90.4)	89.3 (87.0-91.2)	81.2 (80.8-81.6)	80.6 (79.7-81.5)
5-year	70.4 (67.6-68.6)	68.9 (67.8-69.9)	77.3 (75.7-78.8)	78.0 (75.0-80.6)	67.3 (66.8-67.8)	67.8 (66.7-68.9)
10-year	63.2 (60.5-61.6)	62.3 (61.1-63.4)	71.8 (70.1-73.5)	70.9 (67.5-73.9)	60.1 (59.6-60.7)	61.3 (60.1-62.5)
Median (months)	-	-	-	-	-	-
**Distant**						
1-year	49.7 (49.1-50.4)	64.1 (62.8-65.4)	65.5 (63.4-67.6)	80.4 (77.6-83.0)	48.0 (47.3-48.8)	60.8 (59.3-62.3)
2-year	28.7 (28.0-29.3)	44.5 (43.1-45.9)	37.9 (35.8-40.0)	59.0 (55.6-62.3)	27.7 (27.0-28.3)	41.5 (40.0-43.0)
5-year	11.4 (10.5-11.4)	25.8 (24.5-27.1)	15.1 (13.6-16.8)	38.7 (35.3-42.1)	10.5 (10.1-11.0)	23.1 (21.8-24.5)
10-year	7.6 (7.2-8.0)	18.4 (17.2-19.7)	11.0 (9.6-12.6)	27.3 (23.9-30.8)	7.2 (6.8-7.7)	16.5 (15.2-17.9)
Median (months)	11.9	20.0	17.9	32.9	11.2	17.7
**Transverse Colon**						
**Localized**						
1-year	94.5 (94.0-95.0)	95.6 (94.1-96.8)	99.3 (97.9-99.8)	100 (-)	94.2 (93.6-94.7)	95.2 (93.6-96.5)
2-year	92.3 (91.7-93.0)	93.3 (91.5-94.7)	99.1 (97.6-99.7)	98.8 (91.5-99.8)	91.9 (91.2-92.5)	92.8 (90.8-94.3)
5-year	87.2 (86.3-88.0)	87.6 (85.2-89.6)	95.3 (92.8-97.0)	94.7 (86.5-98.0)	86.6 (85.7-87.4)	86.9 (84.3-89.1)
10-year	81.9 (80.8-82.9)	82.0 (79.0-84.7)	90.2 (86.4-92.9)	93.2 (84.3-97.1)	81.3 (80.1-82.4)	80.9 (77.7-83.7)
Median (months)	-	-	-	-	-	-
**Regional**						
1-year	89.2 (88.6-89.9)	90.0 (88.3-91.4)	94.6 (92.7-95.9)	96.1 (92.1-98.1)	88.7 (88.0-89.4)	89.1 (87.3-90.7)
2-year	83.1 (82.2-83.9)	82.3 (80.2-84.2)	89.6 (87.2-91.5)	90.0 (84.6-93.6)	82.4 (81.5-83.2)	81.1 (78.8-83.2)
5-year	70.6 (69.5-71.6)	70.2 (67.6-72.5)	77.6 (74.4-80.4)	76.1 (68.9-81.8)	69.8 (68.7-70.9)	69.3 (66.6-71.9)
10-year	62.8 (61.6-63.9)	63.4 (60.6-66.1)	69.7 (66.1-73.0)	69.0 (61.0-75.7)	62.0 (68.7-70.9)	62.7 (59.6-65.6)
Median (months)	-	-	-	-	-	-
**Distant**						
1-year	51.2 (49.6-52.8)	52.3 (48.2-56.2)	66.6 (62.1-70.6)	66.9 (57.3-74.7)	49.1 (47.4-50.7)	48.9 (44.4-53.3)
2-year	31.7 (30.2-33.2)	30.8 (27.1-34.6)	44.6 (40.1-49.1)	43.4 (34.0-52.3)	29.9 (28.4-31.5)	27.9 (23.9-32.0)
5-year	12.1 (11.0-13.2)	15.0 (12.2-18.1)	22.2 (18.5-26.2)	21.1 (14.0-29.2)	10.6 (9.5-11.8)	13.5 (10.6-16.9)
10-year	8.1 (7.2-9.1)	10.6 (8.0-13.6)	17.1 (13.6-20.9)	11.6 (6.1-19.2)	6.8 (5.8-7.8)	10.6 (7.8-13.9)
Median (months)	12.6	13.1	20.5	20.8	11.6	11.4
**Left Colon**						
**Localized**						
1-year	95.4 (95.2-95.7)	95.9 (95.0-96.7)	99.3 (98.9-99.6)	98.3 (95.4-99.3)	95.0 (94.8-95.3)	95.6 (94.6-96.5)
2-year	93.2 (92.8-93.5)	93.4 (92.3-94.4)	98.3 (97.7-98.8)	97.8 (94.8-99.1)	92.6 (92.3-92.9)	92.9 (91.6-94.0)
5-year	86.9 (86.5-87.3)	86.3 (84.6-87.7)	93.5 (92.4-94.4)	92.8 (88.4-95.5)	86.2 (85.7-86.7)	85.4 (83.7-87.0)
10-year	80.5 (79.9-81.0)	79.8 (77.8-81.7)	87.9 (86.4-89.3)	88.3 (82.9-92.0)	79.2 (79.1-80.2)	78.6 (76.4-80.7)
Median (months)	-	-	-	-	-	-
**Regional**						
1-year	93.0 (92.7-93.2)	91.0 (89.9-91.9)	98.0 (97.5-98.3)	96.1 (93.9-97.5)	92.2 (91.9-92.5)	90.1 (88.9-91.1)
2-year	87.8 (87.4-88.1)	83.3 (82.0-84.6)	94.3 (93.6-95.0)	88.9 (85.7-91.5)	86.7 (86.3-87.1)	82.4 (80.9-83.7)
5-year	73.7 (73.2-74.2)	66.4 (64.7-68.1)	81.1 (79.9-82.2)	70.8 (66.3-74.8)	72.5 (72.0-73.1)	65.7 (63.8-67.5)
10-year	63.2 (62.7-63.8)	57.0 (55.0-58.8)	70.0 (68.5-71.5)	63.4 (58.5-67.8)	62.1 (61.5-62.8)	55.8 (53.7-57.9)
Median (months)	-	-	-	-	-	-

**Distant**						
1-year	63.7 (63.1-64.3)	59.0 (56.9-61.1)	79.0 (77.7-80.3)	70.1 (65.1-74.5)	60.6 (59.9-61.3)	56.6 (54.3-59.0)
2-year	44.8 (44.1-45.4)	36.7 (34.6-38.8)	57.3 (55.6-58.9)	42.6 (37.5-47.7)	42.2 (41.5-42.9)	35.4 (33.1-37.7)
5-year	18.5 (18.0-19.1)	14.6 (13.1-16.3)	23.0 (21.6-24.4)	17.4 (13.6-21.6)	17.6 (17.0-18.2)	14.0 (12.3-15.8)
10-year	11.3 (10.8-11.8)	10.2 (8.8-11.7)	14.4 (13.1-15.8)	13.3 (9.9-17.3)	10.7 (10.1-11.2)	9.5 (8.0-11.2)
Median (months)	20.3	16.5	27.7	20.4	18.5	15.5
**Rectal**						
**Localized**						
1-year	95.0 (94.7-95.2)	94.8 (93.6-95.8)	98.8 (98.4-99.1)	95.7 (91.6-97.8)	94.5 (94.2-94.7)	94.7 (93.3-95.7)
2-year	91.7 (91.3-92.0)	90.1 (88.5-91.5)	97.1 (96.5-97.6)	91.9 (87.0-95.1)	90.9 (90.6-91.3)	89.9 (88.1-91.4)
5-year	82.9 (82.5-83.4)	76.8 (74.5-78.9)	89.8 (88.7-90.8)	84.5 (78.4-89.0)	82.0 (81.5-82.5)	75.7 (73.2-78.0)
10-year	75.1 (74.5-75.7)	68.3 (65.6-70.8)	83.2 (81.8-84.6)	77.7 (70.6-83.3)	74.0 (73.3-74.6)	66.9 (64.0-69.6)
Median (months)	-	-	-	-	-	-
**Regional**						
1-year	93.2 (92.9-93.4)	92.0 (91.0-92.9)	98.0 (97.6-98.3)	94.5 (92.4-96.0)	92.2 (91.9-92.5)	91.4 (90.3-92.4)
2-year	86.6 (86.2-86.9)	80.0 (78.6-81.3)	92.9 (92.3-93.6)	84.0 (81.0-86.6)	85.3 (84.9-85.7)	79.0 (77.4-80.5)
5-year	69.8 (69.3-70.3)	59.4 (57.6-61.1)	78.6 (77.5-79.6)	63.7 (59.8-67.3)	68.0 (67.4-68.5)	58.3 (56.4-60.2)
10-year	57.9 (57.3-58.5)	49.7 (47.8-51.5)	66.6 (65.2-67.9)	53.5 (49.1-57.3)	56.1 (55.4-56.7)	48.8 (46.7-50.8)
Median (months)	-	114	-	-	-	104
**Distant**						
1-year	61.9 (61.2-62.6)	60.3 (57.6-62.9)	75.4 (73.9-76.8)	72.4 (66.6-77.3)	58.9 (58.1-59.6)	57.2 (54.2-60.2)
2-year	39.8 (39.8-40.6)	37.2 (34.6-39.9)	51.5 (49.8-53.2)	44.8 (38.7-50.7)	37.2 (36.4-38.0)	35.3 (32.3-38.2)
5-year	13.3 (12.8-13.8)	13.7 (11.8-15.7)	18.6 (17.2-20.0)	16.1 (11.9-20.9)	12.1 (11.5-12.6)	13.1 (11.0-15.3)
10-year	7.5 (7.0-8.0)	8.1 (6.5-9.9)	10.7 (9.5-11.9)	11.3 (7.6-15.9)	6.8 (6.3-7.3)	7.2 (5.6-9.2)
Median (months)	18.1	16.3	24.8	21.2	16.6	15.1

CI, confidence interval; IN, insufficient to calculate. Right colon includes appendix, cecum, ascending colon, and hepatic flexure. Left colon includes splenic flexure, descending colon, and sigmoid. Rectal includes rectosigmoid colon and rectum.

Table S5. Relative survival of colorectal conventional adenocarcinomas and mucinous adenocarcinomas by age group and site.

Colorectal Location	All Patients		Age < 50 years		Age ≥50 years	
**Relative Survival % (95% CI)**	**Adenocarcinoma**	**Mucinous**	**Adenocarcinoma**	**Mucinous**	**Adenocarcinoma**	**Mucinous**
**All Sites**						
**Localized**						
1-year	94.0 (93.8-94.2)	94.7 (94.1-95.2)	98.7 (98.4-99.0)	98.4 (97.4-99.1)	93.6 (93.4-93.8)	94.3 (93.7-94.8)
2-year	92.1 (91.8-92.3)	92.9 (92.2-93.6)	97.2 (96.8-97.6)	95.8 (94.3-96.9)	91.6 (91.4-91.9)	92.6 (91.8-93.3)
5-year	86.4 (86.0-86.7)	86.9 (85.8-87.9)	91.1 (90.3-91.7)	89.5 (87.3-91.4)	86.0 (85.6-86.3)	86.6 (85.4-87.7)
10-year	79.4 (78.9-79.9)	79.1 (77.5-80.7)	85.0 (84.0-85.9)	84.2 (81.4-86.7)	78.9 (78.3-79.4)	78.5 (76.7-80.2)
Median (months)	-	-	-	-	-	-
**Regional**						
1-year	90.7 (90.5-90.9)	89.9 (89.4-90.4)	97.1 (96.8-97.4)	95.6 (94.6-96.4)	89.8 (89.6-90.0)	89.1 (88.5-89.6)
2-year	84.7 (84.4-84.9)	81.4 (80.7-82.1)	91.9 (91.5-92.4)	87.0 (85.5-88.4)	83.6 (83.4-83.9)	80.6 (79.8-81.3)
5-year	70.9 (70.6-71.3)	67.2 (66.3-68.1)	78.1 (77.4-78.8)	70.4 (68.4-72.4)	69.9 (69.6-70.3)	66.7 (65.7-67.7)
10-year	61.1 (60.7-61.5)	59.2 (58.0-60.3)	67.6 (66.8-68.5)	61.9 (59.6-64.1)	60.2 (59.7-60.7)	58.8 (57.5-60.1)
Median (months)	-	-	-	-	-	-
**Distant**						
1-year	56.8 (56.5-57.2)	60.9 (59.9-61.9)	73.5 (72.6-74.4)	75.2 (73.0-77.2)	54.0 (53.6-54.4)	57.8 (56.7-59.0)
2-year	36.6 (36.2-37.0)	40.3 (39.3-41.3)	49.8 (48.8-50.8)	51.2 (48.7-53.7)	34.4 (34.0-34.8)	37.9 (36.8-39.1)
5-year	13.8 (13.5-14.1)	20.5 (19.6-21.4)	19.1 (18.3-19.9)	27.8 (25.6-30.0)	12.9 (12.6-13.2)	18.9 (17.9-19.9)
10-year	8.6 (8.3-8.8)	14.3 (13.4-15.2)	11.9 (11.1-12.6)	19.6 (17.5-21.9)	8.0 (7.7-8.3)	13.1 (12.1-14.1)
Median (months)	15.6	17.5	23.9	24.8	14.0	15.9
**Right Colon**						
**Localized**						
1-year	94.1 (93.8-94.4)	95.0 (94.2-95.7)	98.8 (98.1-99.3)	99.5 (97.9-99.9)	93.9 (93.5-94.2)	94.6 (93.8-95.3)
2-year	92.8 (92.4-93.2)	93.8 (92.8-94.6)	97.3 (96.4-98.0)	96.5 (94.4-97.8)	92.6 (92.2-93.0)	93.5 (92.5-94.4)
5-year	88.9 (88.3-89.5)	89.4 (88.0-90.7)	93.1 (91.7-94.3)	91.0 (87.9-93.4)	88.7 (88.0-89.3)	89.3 (87.7-90.7)
10-year	83.4 (82.4-84.4)	82.1 (79.7-84.2)	88.4 (86.3-90.1)	85.1 (80.9-88.5)	83.1 (82.1-84.2)	81.8 (79.2-84.0)
Median (months)	-	-	-	-	-	-
**Regional**						
1-year	88.7 (88.4-89.1)	89.2 (88.5-90.0)	95.7 (94.9-96.4)	96.4 (94.9-97.5)	88.2 (87.8-88.5)	88.4 (87.6-89.2)
2-year	81.8 (81.3-82.2)	81.6 (80.6-82.6)	88.6 (87.4-89.7)	88.6 (86.2-90.5)	81.2 (80.7-81.7)	80.8 (79.8-81.9)
5-year	69.8 (69.2-70.3)	70.3 (69.0-71.5)	76.1 (74.5-77.7)	76.2 (73.1-79.0)	69.2 (68.6-69.9)	69.6 (68.2-71.0)
10-year	62.6 (61.8-63.4)	63.4 (61.6-65.1)	69.8 (67.9-71.6)	69.2 (65.7-72.5)	62.0 (61.1-62.9)	62.7 (60.7-64.5)
Median (months)	-	-	-	-	-	-
**Distant**						
1-year	48.6 (47.9-49.3)	63.4 (62.0-64.8)	64.7 (62.6-66.8)	80.1 (77.2-82.6)	46.9 (46.2-47.6)	60.0 (58.5-61.5)
2-year	28.0 (27.4-28.6)	44.1 (42.7-45.5)	37.3 (35.2-39.4)	58.4 (55.0-61.7)	27.0 (26.4-27.6)	41.2 (39.6-42.7)
5-year	10.7 (10.3-11.2)	25.9 (24.6-27.2)	14.6 (13.1-16.2)	37.4 (34.1-40.7)	10.3 (9.9-10.8)	23.4 (22.0-24.9)
10-year	7.5 (7.0-7.9)	18.5 (17.1-19.9)	10.3 (8.9-11.9)	26.2 (22.9-29.7)	7.2 (6.7-7.7)	16.8 (15.3-18.4)
Median (months)	11.5	19.7	17.6	32.4	10.7	17.4
**Transverse Colon**						
**Localized**						
1-year	93.0 (92.2-93.7)	95.6 (93.5-97.0)	99.3 (97.5-99.8)	98.9 (90.6-99.9)	92.5 (91.7-93.3)	95.3 (93.0-96.9)
2-year	91.8 (90.8-92.6)	94.6 (91.8-96.5)	99.1 (97.0-99.7)	97.8 (89.9-99.6)	91.3 (90.2-92.2)	94.3 (91.2-96.3)
5-year	86.7 (85.3-88.0)	88.4 (84.2-91.5)	94.6 (91.5-96.6)	92.1 (82.3-96.6)	86.2 (84.6-87.5)	88.0 (83.4-91.4)
10-year	79.8 (77.5-81.8)	81.3 (74.9-86.2)	89.5 (85.1-92.7)	88.3 (75.5-94.6)	79.1 (76.6-81.3)	80.5 (73.6-85.8)
Median (months)	-	-	-	-	-	-
**Regional**						
1-year	87.0 (86.1-87.7)	89.2 (87.2-90.9)	93.8 (91.8-95.3)	96.4 (92.3-98.4)	86.3 (85.4-87.1)	88.1 (85.9-90.0)
2-year	81.5 (80.5-82.4)	81.5 (79.0-83.7)	88.5 (86.0-90.6)	89.5 (83.9-93.2)	80.8 (79.7-81.8)	80.4 (77.6-82.8)
5-year	69.8 (68.5-71.0)	70.2 (66.9-73.1)	75.5 (72.3-78.5)	74.9 (67.6-80.8)	69.2 (67.8-70.5)	69.5 (65.9-72.8)
10-year	60.4 (58.7-62.1)	62.0 (57.8-65.9)	67.5 (63.7-71.0)	64.8 (56.3-72.0)	59.6 (57.7-61.5)	61.6 (56.9-66.0)
Median (months)	-	-	-	-	-	-
**Distant**						
1-year	50.4 (48.8-52.0)	51.6 (47.5-55.6)	66.1 (61.7-70.2)	65.7 (56.1-73.6)	48.2 (46.5-49.9)	48.4 (43.8-52.8)
2-year	31.1 (29.6-32.6)	30.3 (26.5-34.1)	43.9 (39.4-48.3)	42.7 (33.5-51.6)	29.3 (27.7-30.9)	27.4 (23.4-31.6)
5-year	11.8 (10.7-13.0)	14.9 (12.0-18.1)	21.3 (17.6-25.1)	20.5 (13.5-28.4)	10.4 (9.4-11.6)	13.6 (10.5-17.2)
10-year	8.0 (6.9-9.1)	10.4 (7.6-13.8)	15.9 (12.5-19.6)	11.4 (5.9-18.9)	6.8 (5.7-7.9)	10.0 (6.9-13.6)
Median (months)	12.2	12.8	20.0	20.4	11.1	11.2
**Left Colon**						
**Localized**						
1-year	94.0 (93.6-94.3)	94.1 (92.7-95.2)	98.7 (98.2-99.1)	98.0 (94.9-99.3)	93.5 (93.1-93.9)	93.6 (92.1-94.9)
2-year	92.2 (91.8-92.6)	92.0 (90.4-93.4)	97.7 (96.9-98.2)	96.6 (92.9-98.4)	91.6 (91.2-92.1)	91.5 (89.6-93.0)
5-year	86.9 (86.2-87.5)	85.6 (83.1-87.7)	92.2 (91.0-93.3)	90.6 (85.5-94.0)	86.3 (85.6-87.0)	85.0 (82.3-87.3)
10-year	79.8 (78.8-80.7)	78.2 (74.7-81.4)	86.1 (84.3-87.7)	86.5 (80.4-90.8)	79.1 (78.1-80.1)	77.0 (73.0-80.5)
Median (months)	-	-	-	-	-	-
**Regional**						
1-year	91.8 (91.5-92.1)	89.8 (88.5-90.9)	97.7 (97.2-98.1)	95.1 (92.7-96.8)	90.9 (90.5-91.3)	88.8 (87.4-90.1)
2-year	87.1 (86.7-87.5)	82.4 (80.8-83.8)	93.8 (93.0-94.5)	87.6 (84.2-90.3)	86.1 (85.6-86.5)	81.5 (79.7-83.1)
5-year	73.7 (73.2-74.3)	66.0 (64.0-68.0)	80.1 (78.8-81.2)	68.0 (63.3-72.1)	72.7 (72.1-73.4)	65.7 (63.5-67.9)
10-year	63.6 (62.8-64.3)	58.1 (55.5-60.5)	68.8 (67.3-70.3)	60.0 (55.0-64.7)	62.7 (61.9-63.6)	57.7 (54.8-60.5)
Median (months)	-	-	-	-	-	-
**Distant**						

1-year	62.7 (62.0-63.3)	58.6 (56.4-60.7)	78.4 (77.1-79.7)	69.2 (64.2-73.6)	59.5 (58.7-60.2)	56.3 (53.9-58.7)
2-year	44.1 (43.4-44.8)	36.4 (34.3-38.5)	56.8 (55.1-58.4)	42.1 (37.0-47.1)	41.5 (40.8-42.3)	35.1 (32.8-37.5)
5-year	18.1 (17.6-18.7)	14.0 (12.4-15.7)	22.3 (20.9-23.7)	16.9 (13.2-21.0)	17.3 (16.7-17.9)	13.4 (11.7-15.2)
10-year	10.9 (10.3-11.4)	9.9 (8.4-11.5)	13.7 (12.4-15.1)	13.2 (9.8-17.2)	10.3 (9.7-10.9)	9.1 (7.5-10.9)
Median (months)	19.8	16.4	27.5	20.1	18.0	15.5
**Rectal**						
**Localized**						
1-year	94.1 (93.8-94.4)	93.5 (91.8-94.8)	98.6 (98.0-98.9)	95.5 (91.2-97.7)	93.5 (93.2-93.9)	93.2 (91.4-94.6)
2-year	91.2 (90.7-91.6)	89.2 (87.1-91.0)	96.6 (95.8-97.2)	92.0 (86.9-95.2)	90.5 (90.0-90.9)	88.8 (86.5-90.8)
5-year	83.0 (82.4-83.6)	77.5 (74.6-80.2)	88.7 (87.5-89.8)	82.9 (76.3-87.8)	82.3 (81.6-82.9)	76.8 (73.6-79.7)
10-year	74.6 (73.7-75.5)	67.5 (63.7-71.0)	81.7 (80.1-83.2)	76.3 (68.6-82.3)	73.6 (72.6-74.5)	66.1 (61.8-70.0)
Median (months)	-	-	-	-	-	-
**Regional**						
1-year	92.5 (92.2-92.8)	92.1 (91.0-93.0)	97.8 (97.3-98.1)	94.5 (92.4-96.0)	91.5 (91.1-91.8)	91.5 (90.2-92.6)
2-year	86.1 (85.7-86.5)	79.9 (78.4-81.3)	92.6 (91.9-93.2)	83.8 (80.7-86.4)	84.8 (84.4-85.3)	79.0 (77.2-80.6)
5-year	69.9 (69.4-70.5)	59.6 (57.7-61.4)	77.9 (76.8-79.0)	63.2 (59.3-66.9)	68.3 (67.7-68.9)	58.7 (56.5-60.8)
10-year	57.7 (56.9-58.4)	49.2 (46.9-51.4)	65.5 (64.1-66.9)	52.5 (48.2-56.6)	56.0 (55.2-56.8)	48.4 (45.7-51.0)
Median (months)	-	110	-	-	-	103
**Distant**						
1-year	61.0 (60.3-61.7)	59.4 (56.7-62.0)	74.6 (73.1-76.0)	71.8 (66.0-76.8)	57.9 (57.1-58.7)	56.2 (53.1-59.2)
2-year	39.1 (38.4-39.8)	36.7 (34.1-39.4)	50.8 (49.1-52.5)	44.8 (38.7-50.6)	36.4 (35.6-37.2)	34.6 (31.7-37.6)
5-year	12.8 (12.3-13.3)	13.2 (11.3-15.2)	18.1 (16.8-19.5)	15.5 (11.4-20.2)	11.6 (11.0-12.1)	12.6 (10.5-14.8)
10-year	7.3 (6.8-7.7)	7.3 (5.7-9.1)	10.1 (9.0-11.4)	11.1 (7.4-15.6)	6.6 (6.1-7.1)	6.3 (10.5-14.8)
Median (months)	17.7	15.8	24.4	21.3	16.1	14.7

CI, confidence interval; IN, insufficient to calculate. Right colon includes appendix, cecum, ascending colon, and hepatic flexure. Left colon includes splenic flexure, descending colon, and sigmoid. Rectal includes rectosigmoid colon and rectum.

Table S6. Distribution of gastric conventional adenocarcinomas and mucinous adenocarcinomas by location.

Gastric Location	Adenocarcinoma	Mucinous
**All Sites**	44,239 (100)	1,694 (100)
**Proximal**	29,519 (66.7)	1,057 (62.4)
Cardia	21,828 (49.3)	795 (46.9)
Fundus	2,454 (5.5)	80 (4.7)
Body	5,237 (11.8)	182 (10.7)
**Distal**	14,720 (33.3)	637 (37.6)
Antrum	12,783 (28.9)	556 (32.8)
Pylorus	1,937 (4.4)	8 (4.8)

$p < 0.05$ for all comparisons between conventional adenocarcinomas and mucinous adenocarcinomas between proximal and distal stomach comparisons.

Table S7. Distribution of gastric cancer by localization, dichotomized by sex and age groupings.

Gastric Location	Adenocarcinoma				Mucinous			
**Sex**	**Male**		**Female**		**Male**		**Female**	
**Age (Years)**	**<50**	**≥50**	**<50**	**≥50**	**<50**	**≥50**	**<50**	**≥50**
**All Sites**								
N (%)	2,782 [9.2] (100)	27,380 [90.8] (100)	1086 [7.7] (100)	12,991 [92.3] (100)	120 [10.3] (100)	1,040 [89.7] (100)	48 [9.0] (100)	486 [91.0] (100)
Stage (%)								
In Situ	9 (0.3)	214 (0.8)	5 (0.5)	120 (0.9)	0 (0)	0 (0)	0 (0)	0 (0)
Localized	408 (14.7)	6,711 (24.5)	181 (16.7)	3,533 (27.2)	15 (12.5)	213 (20.5)	10 (20.8)	100 (20.6)
Regional	899 (32.3)	8,308 (30.3)	367 (33.8)	3,622 (27.9)	44 (36.7)	424 (40.8)	13 (27.1)	185 (38.1)
Distant	1,354 (48.7)	9,842 (35.9)	501 (46.1)	4,033 (31.0)	57 (47.5)	335 (32.2)	20 (41.7)	158 (32.5)
Unstaged	114 (4.1)	2,305 (8.4)	32 (2.9)	1,683 (13.0)	4 (3.3)	68 (6.5)	5 (10.4)	43 (8.8)
Incidence	4.12 (3.95–4.29)	135 (133–137)	1.55 (1.45–1.66)	44.5 (43.7–45.3)	0.17 (0.14-0.21)	5.14 (4.83-5.47)	0.07 (0.05-0.10)	1.71 (1.56-1.88)
**Proximal**								
N (%)	2,118 (76.1)	19,806 (72.3)	613 (56.4)	6,982 (53.7)	86 (71.7)	731 (70.3)	24 (50.0)	216 (44.4)
Stage (%)								
In Situ	6 (0.3)	128 (0.6)	2 (0.3)	60 (0.9)	0 (0)	0 (0)	0 (0)	0 (0)
Localized	290 (13.7)	4,697 (13.7)	102 (16.6)	1,798 (25.8)	11 (12.8)	139 (19.0)	4 (16.7)	34 (15.7)
Regional	637 (30.1)	5,684 (28.7)	184 (30.0)	1,699 (24.3)	31 (36.0)	289 (39.5)	5 (20.8)	74 (34.3)
Distant	1,090 (51.5)	7,616 (38.5)	310 (50.6)	2,493 (35.7)	40 (46.5)	245 (34.1)	12 (50.0)	81 (37.5)
Unstaged	95 (4.5)	1681 (8.5)	15 (2.4)	932 (13.3)	4 (4.7)	54 (7.4)	3 (12.5)	27 (12.5)
Incidence	3.35 (3.20–3.50)	104 (103–106)	0.98 (0.90–1.07)	27.0 (26.4–27.6)	0.12 (0.09-0.15)	3.65 (3.39-3.93)	0.04 (0.02-0.06)	0.86 (0.75-0.98)
**Distal**								
N (%)	664 (23.9)	7574 (27.7)	473 (43.6)	6009 (46.3)	34 (28.3)	309 (29.7)	24 (50.0)	270 (55.6)
Stage (%)								
In Situ	1 (0.2)	86 (1.1)	3 (0.6)	60 (1.0)	0 (0)	0 (0)	0 (0)	0 (0)
Localized	118 (178)	2,014 (26.6)	79 (16.7)	1,735 (28.9)	4 (11.8)	74 (23.9)	6 (25.0)	66 (24.4)
Regional	262 (39.5)	2,624 (34.6)	183 (38.7)	1,923 (32.0)	13 (38.2)	135 (43.7)	8 (33.3)	111 (41.1)
Distant	264 (39.8)	2,226 (29.4)	191 (40.4)	1,540 (25.6)	17 (50.0)	86 (27.8)	8 (33.3)	77 (28.5)
Unstaged	19 (2.9)	624 (8.2)	17 (3.6)	751 (12.5)	0 (0)	14 (4.5)	2 (8.3)	16 (5.9)
Incidence	0.77 (0.70–0.85)	30.9 (30.1–31.7)	0.56 (0.50–0.63)	17.5 (17.0–18.0)	0.05 (0.04-0.08)	1.49 (1.32-1.67)	0.03 (0.02-0.05)	0.86 (0.75-0.98)

$p < 0.05$ for all comparisons between conventional adenocarcinomas and signet ring cell adenocarcinomas. [ ] indicates percentages across the age groups within each sex; ( ) indicates percentages within each column. Incidence rates are expressed per 1 million.

Table S8. Derived univariate and multivariable Cox-proportional hazard ratios of mortality for gastric mucinous adenocarcinomas versus conventional adenocarcinomas.

Gastric Location	Mucinous vs. Adenocarcinoma (All Ages)		Mucinous vs. Adenocarcinoma (Age < 50)		Mucinous vs. Adenocarcinoma (Age ≥ 50)	
**HR (95% CI)**	**Univariate**	**Multivariable**	**Univariate**	**Multivariable**	**Univariate**	**Multivariable**
**All Sites**	1.05 (0.99-1.12)	1.13 (1.06-1.20)	1.00 (0.83-1.21)	1.09 (0.91-1.32)	1.05 (0.99-1.13)	1.13 (1.06-1.20)
**Proximal**	1.10 (1.02-1.17)	1.17 (1.08-1.26)	1.06 (0.85-1.33)	1.18 (0.95-1.48)	1.11 (1.02-1.20)	1.16 (1.07-1.26)
Cardia	1.10 (1.01-1.20)	1.15 (1.06-1.25)	1.07 (0.83-1.38)	1.13 (0.88-1.45)	1.10 (1.01-1.21)	1.16 (1.06-1.26)
Fundus	1.13 (0.86-1.48)	1.19 (0.91-1.56)	1.02 (0.50-2.07)	0.71 (0.32-1.57)	1.16 (0.87-1.55)	1.19 (0.88-1.59)
Body	1.09 (0.90-1.32)	1.18 (0.97-1.44)	1.08 (0.57-2.02)	1.63 (0.83-3.20)	1.09 (0.89-1.33)	1.16 (0.95-1.42)
**Distal**	1.00 (0.90-1.12)	1.07 (0.96-1.19)	0.92 (0.65-1.29)	0.93 (0.66-1.31)	1.01 (0.91-1.13)	1.09 (0.98-1.22)
Antrum	1.01 (0.90-1.13)	1.05 (0.94-1.18)	0.91 (0.63-1.31)	0.92 (0.63-1.33)	1.02 (0.91-1.15)	1.07 (0.95-1.21)
Pylorus	0.97 (0.73-1.30)	1.19 (0.89-1.60)	0.92 (0.37-2.27)	0.94 (0.36-2.44)	0.98 (0.72-1.33)	1.19 (0.88-1.63)

$p < 0.05$ for all results unless confidence interval crosses 1. Multivariable adjustment corrected for sex, race, detection stage, grade differentiation, surgery, radiotherapy, and chemotherapy. HR, hazard ratios.

Table S9. Derived univariate and multivariable Cox-proportional hazard ratios of mortality for conventional gastric adenocarcinomas.

Gastric Location	Adenocarcinoma (All Ages)		Adenocarcinoma (Age <50)		Adenocarcinoma (Age ≥50)	
**HR (95% CI)**	**Univariate**	**Multivariable**	**Univariate**	**Multivariable**	**Univariate**	**Multivariable**
**Proximal**	1.00 (Reference)	1.00 (Reference)	1.00 (Reference)	1.00 (Reference)	1.00 (Reference)	1.00 (Reference)
**Distal**	0.79 (0.77-0.81)	0.93 (0.90-0.95)	0.82 (0.75-0.90)	1.01 (0.92-1.11)	0.79 (0.77-0.81)	0.91 (0.88-0.94)
**Cardia**	1.00 (Reference)	1.00 (Reference)	1.00 (Reference)	1.00 (Reference)	1.00 (Reference)	1.00 (Reference)
**Fundus**	1.20 (1.14-1.27)	1.10 (1.04-1.15)	1.11 (0.94-1.32)	1.02 (0.86-1.21)	1.21 (1.15-1.28)	1.10 (1.03-1.16)
**Body**	0.95 (0.92-0.99)	0.96 (0.92-1.00)	1.04 (0.91-1.18)	1.03 (0.90-1.19)	0.95 (0.91-0.99)	0.94 (0.90-0.99)
**Antrum**	0.79 (0.77-0.81)	0.91 (0.88-0.94)	0.85 (0.77-0.94)	0.99 (0.89-1.11)	0.79 (0.76-0.81)	0.89 (0.86-0.92)
**Pylorus**	0.82 (0.77-0.88)	0.91 (0.85-0.97)	0.73 (0.59-0.90)	0.93 (0.74-1.17)	0.83 (0.78-0.89)	0.90 (0.84-0.96)

$p < 0.05$ for all results unless confidence interval crosses 1. Multivariable adjustment corrected for sex, race, detection stage, grade differentiation, surgery, radiotherapy, and chemotherapy. HR, hazard ratios.

Table S10. Derived univariate and multivariable Cox-proportional hazard ratios of mortality for gastric mucinous adenocarcinomas.

Gastric Location	Mucinous (All Ages)		Mucinous (Age <50)		Mucinous (Age ≥50)	
**HR (95% CI)**	**Univariate**	**Multivariable**	**Univariate**	**Multivariable**	**Univariate**	**Multivariable**
**Proximal**	1.00 (Reference)	1.00 (Reference)	1.00 (Reference)	1.00 (Reference)	1.00 (Reference)	1.00 (Reference)
**Distal**	0.71 (0.62-0.80)	0.83 (0.72-0.96)	0.67 (0.45-1.01)	0.59 (0.36-0.97)	0.71 (0.62-0.81)	0.84 (0.73-0.98)
**Cardia**	1.00 (Reference)	1.00 (Reference)	1.00 (Reference)	1.00 (Reference)	1.00 (Reference)	1.00 (Reference)
**Fundus**	1.29 (0.98-1.70)	1.24 (0.93-1.65)	1.05 (0.50-2.20)	1.04 (0.39-2.80)	1.35 (1.00-1.82)	1.25 (0.91-1.71)
**Body**	0.94 (0.77-1.16)	1.01 (0.82-1.26)	1.01 (0.52-1.98)	1.19 (0.55-2.56)	0.93 (0.75-1.15)	1.19 (0.55-2.56)
**Antrum**	0.71 (0.61-0.81)	0.84 (0.71-0.98)	0.69 (0.44-1.07)	0.69 (0.39-1.22)	0.71 (0.61-0.82)	0.84 (0.71-0.99)
**Pylorus**	0.71 (0.53-0.96)	1.00 (0.72-1.37)	0.66 (0.26-1.65)	0.75 (0.24-2.33)	0.72 (0.53-0.99)	0.99 (0.71-1.39)

$p < 0.05$ for all results unless confidence interval crosses 1. Multivariable adjustment corrected for sex, race, detection stage, grade differentiation, surgery, radiotherapy, and chemotherapy. HR, hazard ratios.

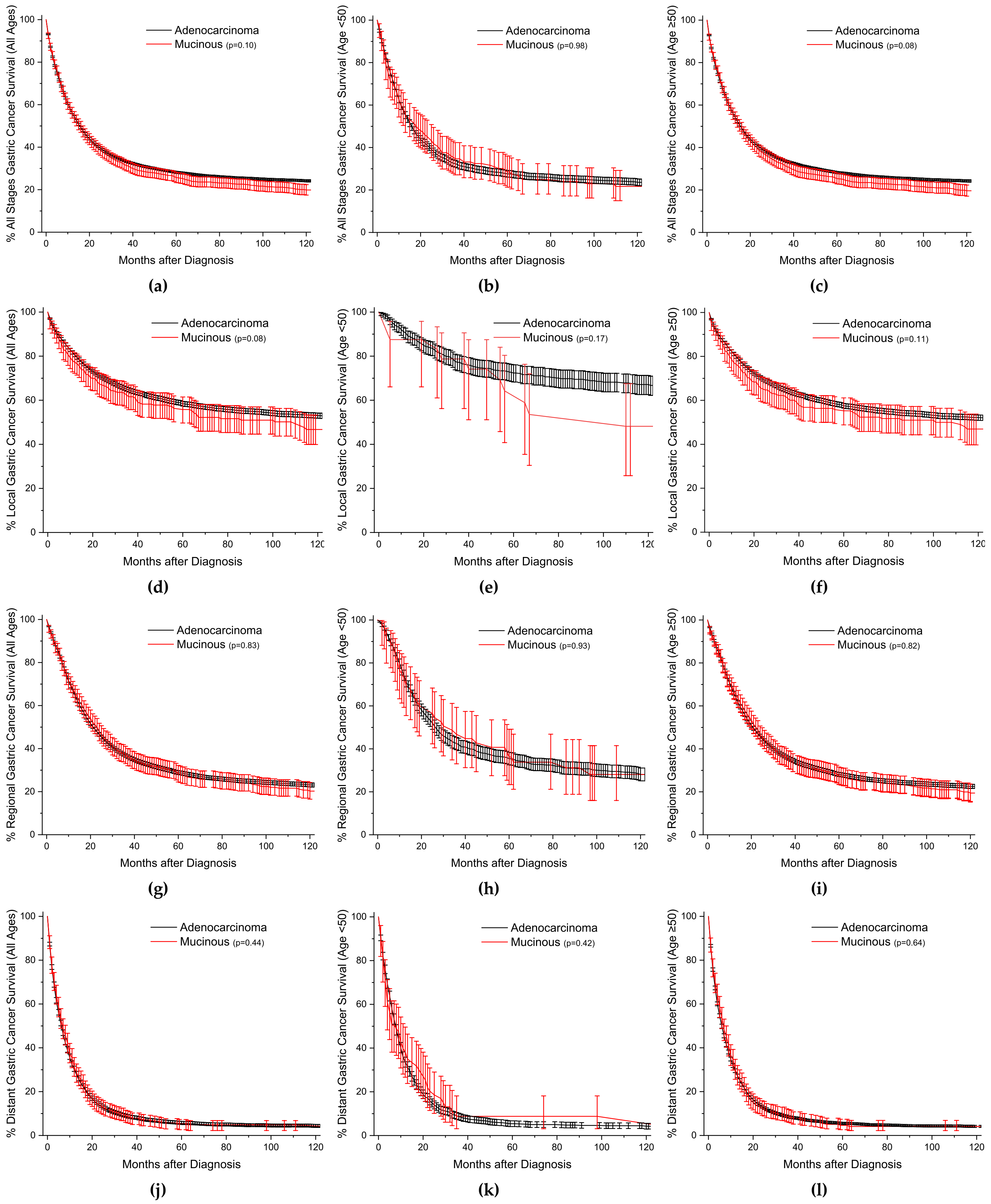

Figure S1. Kaplan-Meier survival curves for gastric cancer. All survivor functions are shown with 95% confidence intervals. (**a**) All stages, all ages. (**b**) All stages, age < 50. (**c**) All stages, age ≥ 50. (**d**) Local disease, all ages, (**e**) Local disease, age < 50. (**f**) Local disease age ≥ 50. (**g**) Regional disease, all ages. (**h**) Regional disease, age < 50. (**i**) Regional disease, age ≥ 50. (**j**) Distant disease, all ages. (**k**) Distant disease, age < 50. (**l**) Distant disease, age ≥ 50. *p*-values between curves by log-rank test.

Table S11. Cause-specific survival of gastric conventional adenocarcinomas and mucinous adenocarcinomas by age group and site.

Gastric Location	All Patients		Age < 50 years		Age ≥ 50 years	
**Cause-Specific Survival % (95% CI)**	**Adenocarcinoma**	**Mucinous**	**Adenocarcinoma**	**Mucinous**	**Adenocarcinoma**	**Mucinous**
**All Sites**						
**Localized**						
1-year	76.8 (75.7-77.8)	74.4 (68.1-79.7)	87.8 (83.9-90.8)	81.3 (52.5-93.5)	76.1 (75.0-77.2)	73.9 (67.2-79.4)
2-year	66.2 (65.0-67.3)	63.6 (56.7-69.7)	82.0 (77.6-85.7)	81.3 (52.5-93.5)	65.2 (64.0-66.4)	62.1 (54.9-68.5)
5-year	54.3 (53.0-55.5)	51.8 (44.6-58.5)	71.4 (66.3-75.8)	60.6 (32.1-80.2)	53.2 (51.9-54.5)	51.1 (43.6-58.1)
10-year	48.8 (47.4-50.2)	43.7 (35.6-51.6)	67.5 (62.1-72.4)	60.6 (32.1-80.2)	47.6 (46.2-49.0)	42.0 (33.3-50.3)
Median (months)	96.8	67.1	-	-	85.1	64.5
**Regional**						
1-year	66.6 (65.5-67.7)	68.8 (64.2-72.9)	75.6 (72.2-78.6)	78.6 (62.9-88.2)	65.7 (64.5-66.8)	67.8 (62.9-72.1)
2-year	46.2 (45.0-47.3)	51.0 (46.2-55.6)	54.4 (50.6-58.0)	66.5 (50.1-78.6)	45.3 (44.1-46.5)	49.3 (44.2-54.2)
5-year	28.6 (27.5-29.7)	32.3 (27.8-36.9)	36.5 (32.8-40.1)	44.3 (28.9-58.6)	27.7 (26.6-28.9)	31.0 (26.3-35.8)
10-year	22.6 (21.5-23.7)	24.1 (19.7-28.7)	29.3 (25.6-33.0)	35.8 (21.2-50.7)	21.9 (20.7-23.0)	22.7 (18.2-27.6)
Median (months)	20.9	24.7	27.7	40.7	20.3	22.9
**Distant**						
1-year	29.6 (28.7-30.5)	32.5 (27.9-37.2)	34.6 (31.9-37.3)	6.8 (20.6-46.6)	28.9 (28.0-29.9)	32.4 (27.4-37.4)
2-year	13.2 (12.6-13.9)	12.4 (9.3-16.0)	16.2 (14.4-18.3)	5.4 (7.8-28.4)	12.8 (12.1-13.6)	11.8 (8.6-15.6)
5-year	5.0 (4.6-5.5)	5.2 (3.2-7.9)	5.2 (4.0-6.6)	3.9 (2.0-17.3)	5.0 (4.6-5.5)	4.9 (2.9-7.7)
10-year	3.8 (3.4-4.3)	4.2 (2.3-6.9)	3.0 (2.8-5.2)	3.9 (2.0-17.3)	3.8 (3.4-4.3)	3.5 (1.7-6.6)
Median (months)	6.0	6.5	8.4	5.3	5.7	6.7
**Proximal**						
**Localized**						
1-year	76.1 (74.9-77.3)	72.5 (64.0-79.3)	87.1 (82.4-90.6)	72.7 (37.1-90.3)	75.5 (74.2-76.7)	72.4 (63.5-79.6)
2-year	64.0 (62.6-65.4)	58.7 (49.6-66.7)	80.2 (74.8-84.5)	72.7 (37.1-90.3)	63.0 (61.6-64.5)	57.3 (47.7-65.7)
5-year	51.5 (50.0-53.0)	42.5 (33.4-51.4)	70.3 (64.3-75.5)	39.8 (11.0-68.0)	50.3 (48.7-51.9)	42.7 (33.2-51.9)
10-year	45.7 (44.1-47.3)	34.1 (24.4-44.1)	66.3 (59.8-71.9)	39.8 (11.0-68.0)	44.3 (42.6-46.0)	33.4 (23.2-43.9)
Median (months)	69.9	40.6	-	56.2	61.0	40.2
**Regional**						
1-year	68.2 (66.9-69.5)	65.8 (59.8-71.1)	77.1 (73.2-80.5)	78.6 (58.4-89.8)	67.2 (65.8-68.6)	64.4 (58.0-70.0)
2-year	46.9 (45.5-48.3)	48.7 (42.6-54.5)	54.8 (50.3-59.0)	67.7 (47.1-81.7)	46.0 (44.5-47.5)	46.5 (40.1-52.7)
5-year	27.9 (26.6-29.2)	29.1 (23.7-34.8)	35.0 (30.8-39.3)	37.6 (20.0-55.2)	27.1 (25.7-28.5)	28.2 (22.5-34.2)
10-year	21.6 (20.3-23.0)	21.8 (16.6-27.6)	27.2 (23.0-31.5)	33.8 (17.1-51.4)	21.0 (19.6-22.4)	20.4 (14.9-26.4)
Median (months)	21.7	22.3	27.4	36.4	21.0	20.7
**Distant**						
1-year	30.7 (29.7-31.7)	36.1 (20.4-41.8)	36.5 (33.4-39.5)	35.5 (19.4-51.9)	29.9 (28.8-31.0)	36.2 (30.2-42.2)
2-year	13.6 (12.8-14.4)	12.3 (8.7-16.6)	17.2 (14.8-19.7)	19.4 (7.9-34.6)	13.1 (12.3-13.9)	11.4 (7.7-15.8)
5-year	5.0 (4.5-5.6)	3.4 (1.7-6.2)	5.5 (4.1-7.2)	6.5 (1.1-18.6)	5.0 (4.4-5.5)	3.0 (1.3-5.9)
10-year	3.7 (3.2-4.2)	2.6 (1.0-5.5)	4.0 (2.8-5.6)	6.5 (1.1-18.6)	3.6 (3.1-4.2)	1.5 (0.2-5.6)
Median (months)	6.5	7.4	9.0	8.5	6.2	7.3
**Distal**						
**Localized**						
1-year	78.3 (76.4-80.1)	77.3 (67.0-84.7)	89.9 (81.4-94.6)	100 (-)	77.7 (75.8-79.6)	75.9 (65.2-83.7)
2-year	71.3 (69.2-73.3)	71.1 (60.2-79.5)	87.6 (78.7-92.9)	100 (-)	70.5 (68.3-72.6)	69.3 (57.9-78.1)
5-year	60.9 (58.6-63.2)	65.5 (54.2-74.7)	74.5 (63.9-82.5)	100 (-)	60.3 (57.9-62.6)	63.3 (51.5-73.0)
10-year	56.3 (53.8-58.7)	59.3 (46.6-69.9)	71.3 (60.2-79.9)	100 (-)	55.5 (52.9-58.0)	56.2 (42.6-67.7)
Median (months)	-	-	-	-	-	-
**Regional**						
1-year	63.0 (61.0-65.0)	73.8 (66.3-79.8)	71.5 (64.5-77.3)	78.6 (47.2-92.5)	62.2 (60.1-64.3)	73.3 (65.5-79.7)
2-year	44.6 (42.5-46.7)	54.8 (30.2-45.3)	53.4 (46.1-60.2)	64.3 (34.3-83.3)	43.8 (41.6-45.9)	53.9 (45.5-61.6)
5-year	30.1 (42.5-46.7)	37.8 (30.2-45.3)	40.2 (33.1-47.2)	57.1 (28.4-78.0)	29.1 (27.1-31.2)	35.9 (28.1-43.8)
10-year	24.6 (22.7-26.6)	27.9 (20.5-35.8)	34.5 (27.5-41.6)	40.0 (14.5-64.7)	23.6 (21.6-25.7)	26.8 (19.1-35.0)
Median (months)	19.3	27.5	28.4	63.0	18.9	26.3
**Distant**						
1-year	25.9 (24.2-27.7)	23.9 (16.5-32.0)	27.5 (22.1-33.2)	29.4 (10.7-51.1)	25.8 (23.9-27.7)	22.8 (15.0-31.6)
2-year	12.1 (10.8-13.4)	12.8 (7.4-19.7)	12.3 (8.5-16.8)	11.8 (2.0-31.2)	12.0 (10.7-13.5)	13.0 (7.2-20.7)
5-year	5.1 (4.2-6.1)	10.6 (5.7-17.3)	4.0 (2.0-7.2)	11.8 (2.0-31.2)	5.3 (4.3-6.4)	10.5 (5.3-17.8)
10-year	4.3 (3.4-5.3)	9.0 (4.3-15.8)	3.4 (1.5-6.5)	11.8 (2.0-31.2)	4.4 (3.5-5.5)	8.6 (3.7-16.1)
Median (months)	4.5	5.1	6.5	3.5	4.3	5.3

CI, confidence interval; Proximal includes cardia, fundus, and body; Distal includes antrum and pylorus.

Table S12. Relative survival of gastric conventional adenocarcinomas and mucinous adenocarcinomas by age group and site.

Gastric Location	All Patients		Age < 50 years		Age ≥50 years	
**Relative Survival % (95% CI)**	**Adenocarcinoma**	**Mucinous**	**Adenocarcinoma**	**Mucinous**	**Adenocarcinoma**	**Mucinous**
**All Sites**						
**Localized**						
1-year	74.0 (72.9-75.1)	70.3 (63.5-76.2)	86.9 (82.9-90.0)	76.6 (48.8-90.6)	73.3 (72.1-74.4)	69.8 (62.6-75.9)
2-year	63.0 (61.8-64.3)	60.8 (53.3-67.4)	80.4 (75.8-84.2)	76.6 (48.8-90.6)	62.0 (60.7-63.3)	59.4 (51.6-66.3)
5-year	50.4 (49.0-51.8)	46.8 (38.8-54.4)	68.2 (62.9-72.8)	58.0 (30.3-77.9)	49.4 (47.9-50.8)	45.8 (37.5-53.8)
10-year	42.6 (40.8-44.3)	35.7 (26.4-45.2)	62.0 (56.1-67.3)	49.5 (21.2-72.8)	41.4 (39.5-43.2)	34.5 (24.6-44.5)
Median (months)	61.8	41.0	-	113	56.8	40.3
**Regional**						
1-year	65.3 (64.2-66.4)	67.9 (63.2-72.2)	74.7 (71.3-77.8)	78.8 (63.0-88.5)	64.3 (63.1-65.5)	66.8 (61.7-71.3)
2-year	44.6 (43.4-45.8)	50.2 (45.2-55.0)	53.0 (49.3-56.6)	66.9 (50.3-79.0)	43.7 (42.4-44.9)	48.4 (43.2-53.5)
5-year	27.1 (26.0-28.2)	31.2 (26.4-36.0)	34.8 (31.2-38.4)	45.2 (29.4-59.7)	26.3 (25.1-27.5)	29.6 (24.7-34.7)
10-year	20.7 (19.5-22.0)	22.9 (18.1-28.1)	26.7 (23.2-30.4)	34.5 (19.8-49.8)	20.1 (18.8-21.4)	21.6 (16.5-27.1)
Median (months)	19.9	24.2	26.6	41.0	19.2	22.4
**Distant**						
1-year	28.7 (27.8-29.5)	30.8 (26.3-35.4)	34.2 (31.5-36.8)	32.1 (19.7-45.1)	27.9 (27.0-28.9)	30.6 (25.9-35.5)
2-year	12.6 (12.0-13.3)	11.5 (8.6-14.9)	15.9 (13.9-18.0)	16.1 (7.5-27.5)	12.2 (11.5-12.9)	10.8 (7.8-14.4)
5-year	4.6 (4.2-5.1)	4.7 (2.9-7.3)	4.9 (3.8-6.3)	6.1 (1.6-15.0)	4.6 (4.1-5.1)	4.5 (2.6-7.2)
10-year	3.5 (3.0-3.9)	3.5 (1.8-6.1)	3.7 (2.7-5.0)	6.1 (1.6-15.0)	3.4 (3.0-3.9)	2.9 (1.2-5.9)
Median (months)	5.8	6.1	8.3	5.3	5.5	6.3
**Proximal**						
**Localized**						
1-year	73.4 (72.0-74.7)	67.4 (58.4-74.9)	85.9 (81.1-89.5)	66.8 (33.7-86.1)	72.6 (71.2-74.0)	67.5 (58.0-75.3)
2-year	60.8 (59.3-62.3)	54.8 (45.4-63.2)	77.9 (72.4-82.5)	66.8 (33.7-86.1)	59.7 (58.2-61.3)	53.6 (43.7-62.5)
5-year	47.5 (45.9-49.2)	36.1 (27.0-45.2)	66.5 (60.3-71.9)	37.0 (10.3-64.8)	46.3 (44.6-48.1)	35.8 (26.3-45.5)
10-year	39.6 (37.6-41.7)	29.3 (19.1-40.2)	60.5 (53.8-66.6)	37.0 (10.3-64.8)	38.2 (36.1-40.3)	28.3 (17.7-39.8)
Median (months)	48.3	33.6	-	40.9	44.7	29.7
**Regional**						
1-year	66.8 (65.5-68.1)	65.8 (59.7-71.3)	77.0 (73.1-80.4)	78.8 (58.5-90.0)	65.7 (64.3-67.1)	64.4 (57.8-70.2)
2-year	45.1 (43.7-46.6)	48.6 (42.3-54.6)	53.9 (49.4-58.1)	68.0 (47.2-82.0)	44.2 (42.7-45.7)	46.4 (39.7-52.7)
5-year	26.2 (24.9-27.5)	29.7 (23.8-35.8)	33.6 (29.4-37.8)	38.3 (20.3-56.1)	25.3 (23.9-26.7)	28.8 (22.6-35.2)
10-year	19.5 (18.1-21.0)	21.5 (15.4-28.2)	24.7 (20.6-29.0)	34.5 (17.4-52.3)	19.0 (17.4-20.5)	19.7 (13.5-26.9)
Median (months)	20.5	22.5	26.7	36.4	19.9	20.7
**Distant**						
1-year	29.7 (28.7-30.7)	34.2 (28.6-39.8)	35.9 (32.9-38.9)	34.4 (18.8-50.7)	28.9 (27.8-29.9)	34.2 (28.3-40.1)
2-year	13.0 (12.2-13.7)	11.7 (8.3-15.9)	16.8 (32.9-38.9)	18.8 (7.6-33.8)	12.4 (11.7-13.2)	10.8 (7.3-15.1)
5-year	4.7 (4.2-5.2)	3.5 (1.7-6.2)	5.2 (3.9-6.8)	6.3 (1.1-18.3)	4.6 (4.1-5.1)	3.1 (1.4-6.0)
10-year	3.3 (2.9-3.9)	2.6 (0.9-5.7)	3.9 (2.7-5.4)	6.3 (1.1-18.3)	3.3 (2.7-3.9)	1.5 (0.2-5.8)
Median (months)	6.3	7.0	8.9	8.0	5.9	7.0
**Distal**						
**Localized**						
1-year	75.6 (73.4-77.5)	74.7 (63.3-83.0)	90.1 (81.6-94.8)	100 (-)	74.9 (72.7-76.9)	73.2 (61.3-81.9)
2-year	68.5 (66.1-70.7)	70.1 (57.5-79.6)	88.0 (78.9-93.3)	100 (-)	67.6 (65.1-69.9)	68.2 (55.1-78.2)
5-year	57.3 (54.5-60.0)	61.4 (47.3-72.8)	73.3 (62.3-81.6)	100 (-)	56.6 (53.7-59.3)	58.8 (44.2-70.8)
10-year	49.7 (46.0-53.2)	44.9 (27.3-61.1)	66.1 (53.4-76.1)	78.3 (8.5-97.6)	48.8 (45.0-52.4)	41.9 (24.0-58.8)
Median (months)	117	106	-	-	111	101
**Regional**						
1-year	62.0 (59.9-64.0)	71.3 (63.4-77.7)	68.9 (62.0-74.9)	78.9 (47.2-92.8)	61.3 (59.1-63.4)	70.6 (62.3-77.4)
2-year	43.4 (41.3-45.5)	52.8 (44.6-60.4)	50.9 (43.8-57.6)	64.7 (34.4-83.7)	42.7 (40.4-44.9)	51.7 (43.1-59.7)
5-year	29.2 (27.2-31.3)	33.3 (25.6-41.2)	37.8 (31.0-44.6)	57.9 (28.6-78.8)	28.4 (26.2-30.6)	30.8 (22.9-39.0)
10-year	23.3 (21.0-25.7)	24.9 (17.1-33.4)	31.5 (24.8-38.5)	35.6 (11.9-60.6)	22.5 (20.0-25.0)	23.9 (15.7-33.1)
Median (months)	18.6	25.9	26.4	79.2	18.3	24.7
**Distant**						
1-year	25.3 (23.5-27.0)	23.1 (16.0-30.9)	27.6 (22.3-33.2)	27.8 (10.1-49.0)	25.0 (23.2-26.9)	22.3 (14.8-30.7)
2-year	11.6 (10.3-12.9)	10.9 (6.1-17.2)	12.7 (8.9-17.1)	11.2 (1.9-29.9)	11.5 (10.1-12.9)	10.8 (5.7-17.7)
5-year	4.4 (3.6-5.4)	7.8 (3.7-13.7)	3.8 (1.9-6.8)	5.6 (0.4-22.5)	4.5 (3.6-5.5)	8.2 (3.7-14.9)
10-year	3.8 (2.9-4.8)	5.6 (2.1-11.6)	3.2 (1.4-6.2)	5.6 (0.4-22.5)	3.8 (2.9-4.9)	5.3 (1.7-12.2)
Median (months)	4.4	5.1	6.3	4.0	4.1	5.2

CI, confidence interval; Proximal includes cardia, fundus, and body; Distal includes antrum and pylorus.
